# Risk factors for thoracic aortic aneurysm and dissection among diabetic patients: a nationwide population-based study

**DOI:** 10.3389/fcvm.2025.1569886

**Published:** 2025-10-07

**Authors:** Suk Kyung Lim, Kyungdo Han, Jun Ho Lee, Kyu Na Lee, In Young Cho, Sang-Man Jin, Kiick Sung, Yang Hyun Cho, Dong Wook Shin

**Affiliations:** ^1^ Department of Thoracic and Cardiovascular Surgery, Samsung Medical Center, Sungkyunkwan University School of Medicine, Seoul, Republic of Korea; ^2^ Department of Statistics and Actuarial Science, Soongsil University, Seoul, Republic of Korea; ^3^ Department of Thoracic and Cardiovascular Surgery, Korea University Anam Hospital, Korea University College of Medicine, Seoul, Republic of Korea; ^4^ Department of Biomedicine and Health Science, The Catholic University of Korea, Seoul, Republic of Korea; ^5^ Department of Family Medicine & Supportive Care Center, Samsung Medical Center, Sungkyunkwan University School of Medicine, Seoul, Republic of Korea; ^6^ Division of Endocrinology, Department of Medicine, Samsung Medical Center, Sungkyunkwan University School of Medicine, Seoul, Republic of Korea; ^7^ Department of Clinical Research Design and Evaluation/ Department of Digital Health, Samsung Advanced Institute for Health Sciences & Technology (SAIHST), Sungkyunkwan University, Seoul, Republic of Korea

**Keywords:** thoracic aortic dissection and aneurysm, diabetes mellitus, nationwide population-based cohort study, risk factors, metformin

## Abstract

**Background:**

Diabetes mellitus (DM) has been reported to be associated with decreased risk for thoracic aortic aneurysm and dissection (TAAD). However, risk factors for TAAD in diabetic patients remain undetermined. This study aims to investigate diabetes-specific risk factors for TAAD development in diabetic patients.

**Methods:**

This population-based study utilized the National Health Insurance Service database in Republic of Korea. We followed 2,328,347 type 2 DM patients undergoing health check-ups from 2009 to 2012 until new TAAD diagnosis, death, or December 31, 2019. Cox proportional-hazards regression models were used to identify risk factors for TAAD development.

**Results:**

TAAD was newly diagnosed in 0.02% (4,512/2,328,347) of patients. In the fully-adjusted model incorporating baseline characteristics and antidiabetic medications, the risk for TAAD was increased with age (HR: 1.05, 95% CI: 1.05–1.06) and males (HR: 1.37, 95% CI: 1.26–1.49). Meanwhile, the risk of TAAD was decreased in patients with a longer diabetes duration (HR: 0.97, 95% CI: 0.96–0.99) and metformin use (HR: 0.91 95% CI: 0.85–0.97).

**Conclusions:**

Our study findings suggest that longer diabetes duration and metformin may reduce TAAD risk. Additional research is needed to investigate whether changes in glucose control and treatment strategies can decrease the development of TAAD in diabetic patients.

## Introduction

Diabetes mellitus (DM) is a well-established risk factor for atherosclerosis. While atherosclerosis has been reported to be an independent risk factor for aortic aneurysm, numerous studies have demonstrated inverse correlations between DM and thoracic aortic aneurysm and dissection (TAAD) (1–4), as well as abdominal aortic aneurysm (AAA) (5–7). Several pathophysiological mechanisms—such as increased extracellular matrix cross-linking, reduced protease activity, and vascular smooth muscle cell senescence—have been proposed to explain this observation. These alterations may lead to increased stiffness and resistance to dilation in the aortic wall, potentially mitigating aneurysm formation (8). Diabetic patients have been reported to exhibit notably lower average hospitalization rates for TAAD than individuals without DM (9.7 per 10,000 compared to 15.6 per 10,000, respectively) (1). The incidence of in-hospital deaths in diabetic patients who underwent proximal aorta surgery, however, was more than three times higher in those with DM than in those without (9).

Despite the inverse correlation between DM and TAAD, only a few studies have evaluated diabetes-specific factors—such as diabetes duration and antidiabetic medications—in relation AAA (10, 11), and such investigations are notably absent in TAAD**.** Metformin, the most commonly prescribed antidiabetic medication, has recently gained attention for its potential ability to inhibit the growth of AAA through its anti-inflammatory effects (10, 12). However, the impact of metformin on TAAD development has not been widely studied, with only two small-scale studies reporting conflicting results (13, 14).

Therefore, to address this knowledge gap, we sought to investigate diabetes-specific risk factors associated with the development of TAAD using a large, nationwide cohort of diabetic patients in South Korea. Specifically, we aimed to examine the impact of diabetes duration, glycemic control, and antidiabetic medications on TAAD risk, while also accounting for conventional cardiovascular comorbidities.

## Methods

### Data source

The study was approved by the Institutional Review Board (IRB) of Soongsil University (IRB number: SSU-202003-HR-201-01). The requirement for informed consent was waived by the IRB as we performed our analyses using anonymized data.

The National Health Insurance Service (NHIS) in Republic of Korea covers 97% of the Korean population. The NHIS database includes demographic information, diagnoses coded using the Tenth Revision of the International Classification of Diseases (ICD-10), prescriptions, and interventions.

### Patient selection

**Figure 1:**
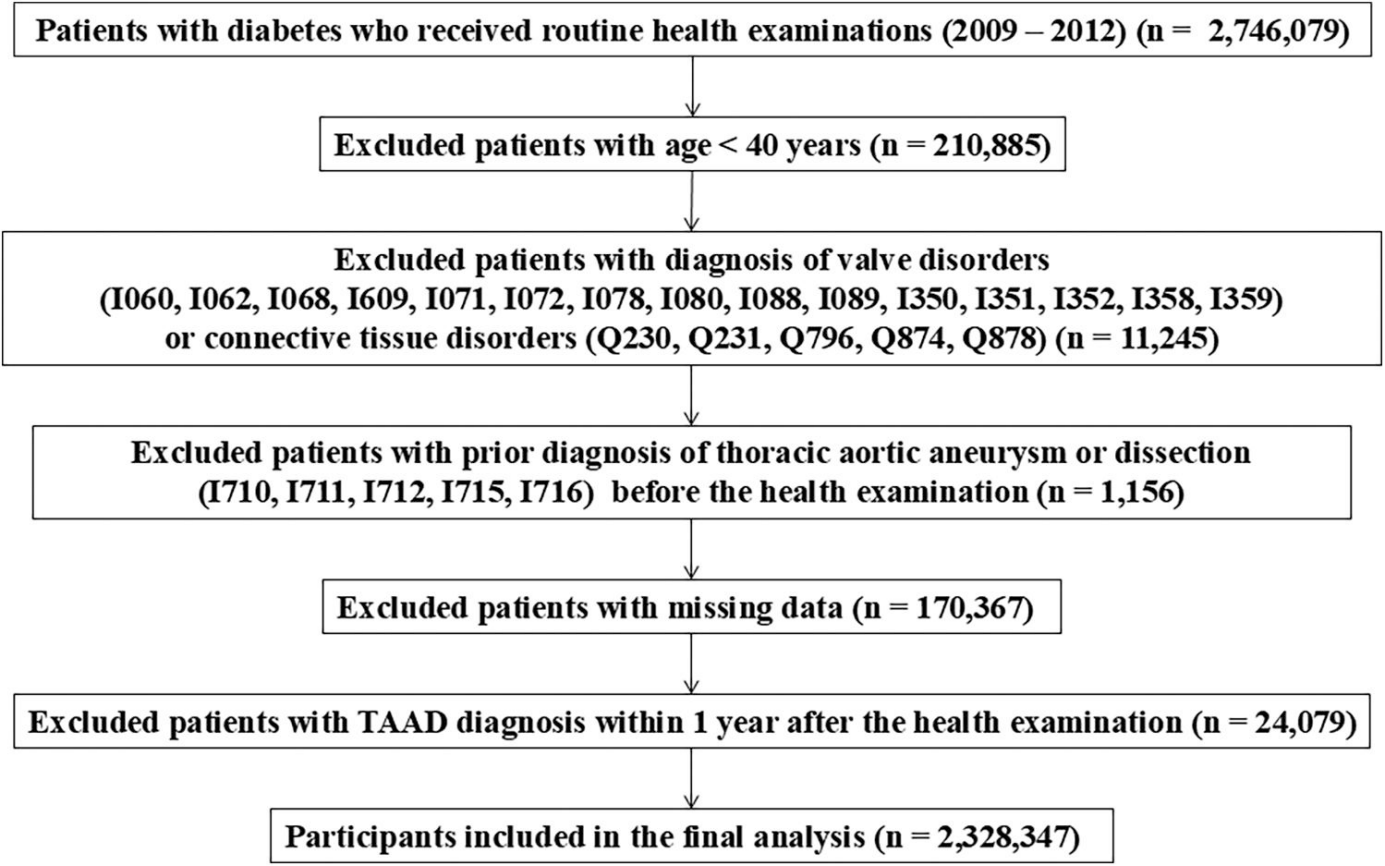
Flow diagram of patients included in the study.

A flow diagram of the study is provided in Figure 1. A total of 2,746,079 patients with type 2 diabetes mellitus (DM) who underwent routine health check-ups between 2009 and 2012 was initially identified. We excluded 210,885 individuals under the age of 40 to minimize the inclusion of patients with genetic conditions such as familial thoracic aortic aneurysm syndromes. To reduce bias from pre-existing aortic or congenital cardiovascular diseases, we further excluded 11,245 patients with relevant ICD-10 diagnoses—such as I060, I062, I068, I609, I071, I072, I078, I080, I088, I089, I350, I351, I352, I358, I359, Q230, Q231, Q796, Q874, and Q878—indicating conditions like valvular disease or connective tissue disorders (e.g., Marfan syndrome) recorded within 3 years prior to the health screening. Additionally, 1,156 patients with a prior diagnosis of aortic disease (ICD-10 codes I710, I711, I712, I715, and I716) were excluded as part of a 3-year washout period. After excluding 170,367 patients with missing key variables and 24,079 with less than one year of follow-up, a total of 2,328,347 patients was included in the final analysis. Although pregnancy and active cancer treatment were not predefined exclusion criteria, the likelihood of including such individuals is considered minimal, as the study population comprised asymptomatic adults aged 40 years or older undergoing routine national health screening.

### Definition of covariates

Hypertension was defined as systolic blood pressure (BP) ≥ 140 mmHg or diastolic BP ≥ 90 mmHg or codes I10–I13 or I15 with a prescription for antihypertensive medication. Dyslipidemia was identified by code E78 and a prescription for lipid-lowering medication or total cholesterol ≥240 mg/dl. Chronic kidney disease (CKD) was defined as codes N18, N19. History of stroke was identified by the codes of I63, I64. Three groups were defined based on smoking status: non-smokers, former smokers, and current smokers. Former and current smokers were further subdivided into those with <20 pack-years (PY) and those ≥20 PY. Subjects were assigned to non-drinker, mild drinker (average alcohol intake <30 g/day), or heavy drinker (average alcohol intake ≥30 g/day) (15). Regular exercise was defined as strenuous activity for ≥20 min ≥3 days per week or moderate activity for ≥30 min ≥5 days per week. Abdominal obesity was defined as a waist circumference ≥90 cm in men and ≥85 cm in women.

### Study outcomes

The primary endpoint of the study was the newly diagnosed composite of thoracic aortic aneurysm (TAA) or thoracic aortic dissection (TAD) or thoracoabdominal aortic aneurysm (TAAA) based on ICD-10 codes; from this point forward, this composite endpoint will be referred to as TAAD. Diagnostic codes for aortic disease were summarized in Supplementary Table S1. Definitions were as follows: (1) ≥2 outpatient visits based on a diagnosis of TAA or TAD or TAAA; (2) ≥1 inpatient admission based on the mentioned diagnosis; or (3) ≥1 record of surgery of the ascending aorta (O2031) or aortic arch (O2032) or descending aorta (O2033) or thoracic endovascular repair (M6611) (16, 17). We followed the study cohort 1 year after the baseline health check-ups to incorporate a 1-year lag period, until the point of new-onset TAAD, death, or December 31, 2019, whichever occurred first.

### Statistical analysis

Statistical analyses were performed using SAS version 9.4 (SAS Institute, Cary, NC). Continuous and categorical variables were compared using independent *t*-tests and chi-square tests, respectively. Cox proportional-hazards regression models were used to identify risk factors for TAAD. First, univariate analyses were employed for each variable. Multivariate analyses included baseline characteristics of age, sex, income, body mass index (BMI), abdominal obesity, smoking, drinking, exercise, hypertension, dyslipidemia, high-density lipoprotein cholesterol (HDL-C), low-density lipoprotein cholesterol (LDL-C), triglycerides (TG), CKD, history of stroke, number of oral hypoglycemic agent (OHA)s, DM duration, fasting blood glucose (FBG) level, use of insulin, metformin, meglitinide, thiazolidinedione, dipeptidyl peptidase 4 inhibitors, alpha-glucosidase inhibitor, and sulfonylurea. Furthermore, the interaction analysis was conducted using multivariable Cox proportional hazards models to test for potential interaction effects between age and sex.

## Results

### Patient characteristics and development of TAAD

New onset TAAD occurred in 0.02% (4,512/2,328,347) of patients during a mean follow-up of 7.7 years. Forty patients died upon hospital arrival. The characteristics of patients are summarized in Table 1. Patients in whom TAAD occurred were older (65.2 vs. 59.4 years, *P* < 0.001); more likely to be male (61.8% vs. 57.9%, *P* < 0.001). Patients who developed TAAD were more likely to be current smokers and non-alcohol drinkers. The proportion of patients with DM duration ≥5 years and insulin use was greater in those who developed TAAD than those who did not. Additional subgroup analyses comparing baseline characteristics between patients with and without TAD, TAA, and TAAA are presented in Supplementary Tables SII–SIV, respectively. Across all three aortic diseases, older age, male sex, hypertension, CKD, and history of stroke were consistently more prevalent. Notably, TAAA patients showed the highest burden of comorbidities, including abdominal obesity (47.5%) and insulin use (12.2%). The proportion of current smokers with ≥20 pack-years was also highest in the TAAA group (20.2%).

**Table 1:** Baseline characteristics of the study population.

Variables	TAAD		
	No (*n* = 2,323,835)	Yes (*n* = 4,512)	*P* value
Age, years	59.4 ± 10.7	65.2 ± 9.7	<.001
Female	978,334 (42.1)	1,722 (38.2)	<.001
Income, lowest quartile	494,704 (21.3)	985 (21.8)	.374
Hypertension	1,377,462 (59.3)	3,465 (76.8)	<.001
Dyslipidemia	1,002,623 (43.2)	2,302 (51.0)	<.001
CKD	284,192 (12.2)	992 (22.0)	<.001
Heart failure	44,264 (1.9)	192 (4.3)	<.001
Coronary heart disease	285,495 (12.3)	1,235 (27.4)	<.001
COPD	214,090 (9.2)	633 (14.0)	<.001
History of stroke	48,458 (2.1)	228 (5.1)	<.001
Smoking			<.001
Never	1,345,076 (57.9)	2,398 (53.2)	
Ex & <20 PY	215,400 (9.3)	361 (8.0)	
Ex & ≥20 PY	215,684 (9.3)	610 (13.5)	
Current & <20 PY	207,452 (8.9)	369 (8.2)	
Current & ≥20 PY	340,223 (14.6)	774 (17.2)	
Drinking			<.001
Non	1,384,763 (59.6)	2,924 (64.8)	
Mild	717,464 (30.9)	1,232 (27.3)	
Heavy	221,608 (9.5)	356 (7.9)	
Regular exercise	489,564 (21.1)	914 (20.3)	.183
BMI, kg/m^2^	25.0 ± 3.3	25.2 ± 3.4	<.001
Abdominal obesity	895,647 (38.5)	2,157 (47.8)	<.001
DM duration, ≥5 years	764,792 (32.9)	1,586 (35.2)	.001
OHA, ≥3	360,934 (15.5)	681 (15.1)	.416
Insulin	229,899 (9.9)	588 (13.0)	<.001
Metformin	1,179,543 (50.8)	2,351 (52.1)	.071
Meglitinide	42,933 (1.9)	109 (2.4)	.005
Thiazolidinedione	159,216 (6.9)	315 (7.0)	.730
DPP-4 inhibitor	215,757 (9.3)	385 (8.5)	.082
Alpha-glucosidase inhibitor	282,543 (12.2)	618 (13.7)	.002
Sulfonylurea	1,025,338 (44.1)	2,162 (47.9)	<.001
Systolic BP, mmHg	129.3 ± 15.9	131.5 ± 16.9	<.001
Fasting glucose, mg/dl	143.8 ± 46.4	136.4 ± 43.7	<.001
Total cholesterol, mg/dl	196.1 ± 42.5	191.3 ± 43.6	<.001
HDL-C, mg/dl	51.9 ± 22.2	50.5 ± 24.2	<.001
LDL-C, mg/dl	111.3 ± 40.8	109.0 ± 42.1	<.001
TG, mg/dl	144.1 [144.0, 144.2]	142.6 [140.4, 144.8]	<.001

Values are presented as the as the mean ± standard deviation or geometric mean [95% confidence interval] for continuous data and as number (%) for categorical data.

BMI, body mass index; BP: blood pressure, CKD, chronic kidney disease; COPD, chronic obstructive pulmonary disease; DM, diabetes mellitus; DPP-4, dipeptidyl peptidase 4; HDL-C, high density lipoprotein cholesterol; LDL-C, low density lipoprotein cholesterol; OHA, oral hypoglycemic agent; PY, pack-years; TAAD, thoracic aortic aneurysm and dissection; TG, triglycerides.

### Independent predictors of the development of TAAD

Significant risk factors for TAAD are presented in Table 2, with the full version, including all variables, provided as Supplementary Table SV. In the fully adjusted model 4 incorporating baseline characteristics, number of OHAs, and antidiabetic medications, the risk of TAAD increased with age (hazard ratio [HR] 1.06, 95% confidence interval [CI] 1.05–1.06), and was higher in male patients (HR: 1.37, 95% CI: 1.26–1.49).

**Table 2:** Multivariate analysis for development of TAAD.

Variables	Model 1^a^	*P* value	Model 2^b^	*P* value	Model 3^c^	*P* value	Model 4^d^	*P* value
Age, year	1.057 (1.053, 1.061)	<.001	1.057 (1.053, 1.061)	<.001	1.057 (1.053, 1.061)	<.001	1.054 (1.051, 1.058)	<.001
Sex		<.001		<.001		<.001		<.001
Male	1.391 (1.279, 1.512)		1.385 (1.273, 1.506)		1.385 (1.274, 1.507)		1.369 (1.259, 1.489)	
Female	1 (Ref.)		1 (Ref.)		1 (Ref.)		1 (Ref.)	
Abdominal obesity		<.001		<.001		<.001		<.001
No	1 (Ref.)		1 (Ref.)		1 (Ref.)		1 (Ref.)	
Yes	1.252 (1.162, 1.349)		1.253 (1.163, 1.350)		1.253 (1.163, 1.350)		1.243 (1.154, 1.340)	
Smoking		<.001		<.001		<.001		<.001
Non	1 (Ref.)		1 (Ref.)		1 (Ref.)		1 (Ref.)	
Ex & <20 PY	1.121 (0.992, 1.265)		1.121 (0.993, 1.266)		1.121 (0.993, 1.266)		1.121 (0.993, 1.266)	
Ex & ≥20 PY	1.526 (1.375, 1.693)		1.530 (1.379, 1.698)		1.529 (1.378, 1.697)		1.509 (1.360, 1.675)	
Current & <20 PY	1.625 (1.440, 1.833)		1.624 (1.440, 1.832)		1.625 (1.440, 1.833)		1.643 (1.457, 1.854)	
Current & ≥20 PY	1.828 (1.656, 2.019)		1.834 (1.660, 2.025)		1.833 (1.660, 2.025)		1.838 (1.665, 2.030)	
Drinking		.002		.001		.002		.007
Non	1 (Ref.)		1 (Ref.)		1 (Ref.)		1 (Ref.)	
Mild	0.897 (0.830, 0.969)		0.894 (0.828, 0.966)		0.895 (0.828, 0.967)		0.907 (0.840, 0.980)	
Heavy	0.828 (0.733, 0.935)		0.825 (0.730, 0.932)		0.826 (0.731, 0.933)		0.848 (0.750, 0.958)	
Hypertension		<.001		<.001		<.001		<.001
No	1 (Ref.)		1 (Ref.)		1 (Ref.)		1 (Ref.)	
Yes	1.726 (1.597, 1.865)		1.733 (1.604, 1.873)		1.731 (1.602, 1.871)		1.639 (1.516, 1.772)	
Dyslipidemia		<.001		<.001		<.001		<.001
No	1 (Ref.)		1 (Ref.)		1 (Ref.)		1 (Ref.)	
Yes	1.294 (1.214, 1.379)		1.305 (1.224, 1.391)		1.302 (1.222, 1.388)		1.223 (1.146, 1.304)	
Heart failure								.003
No							1 (Ref.)	
Yes							1.252 (1.079, 1.452)	
Coronary heart disease								<.001
No							1 (Ref.)	
Yes							1.837 (1.712, 1.972)	
COPD								.002
No							1 (Ref.)	
Yes							1.146 (1.052, 1.249)	
HDL-C, quartile (mg/dl)		<.001		<.001		<.001		<.001
Q1 (<41)	1.239 (1.135, 1.352)		1.243 (1.139, 1.357)		1.242 (1.138, 1.356)		1.215 (1.113, 1.326)	
Q2 (42–49)	1.054 (0.966, 1.149)		1.057 (0.970, 1.153)		1.056 (0.969, 1.152)		1.041 (0.955, 1.135)	
Q3 (50–58)	1.004 (0.920, 1.096)		1.006 (0.922, 1.099)		1.006 (0.921, 1.098)		0.998 (0.914, 1.089)	
Q4 (≥59)	1 (Ref.)		1 (Ref.)		1 (Ref.)		1 (Ref.)	
FBG, mg/dl		<.001		<.001		<.001		<.001
≤130	1 (Ref.)		1 (Ref.)		1 (Ref.)		1 (Ref.)	
>130	0.897 (0.845, 0.952)		0.882 (0.830, 0.937)		0.887 (0.835, 0.943)		0.897 (0.844, 0.953)	
Number of OHAs ≥3		.0212				.063		.165
<3	1 (Ref.)				1 (Ref.)		1 (Ref.)	
≥3	0.903 (0.827, 0.985)				0.877 (0.764, 1.007)		0.907 (0.791, 1.041)	
DM duration, year	0.968 (0.958, 0.978)	<.001	0.973 (0.962, 0.985)	<.001	0.972 (0.960, 0.984)	<.001	0.974 (0.963, 0.985)	<.001
Insulin		<.001		<.001		<.001		.002
No	1 (Ref.)		1 (Ref.)		1 (Ref.)		1 (Ref.)	
Yes	1.248 (1.134, 1.374)		1.232 (1.118, 1.358)		1.234 (1.120, 1.360)		1.166 (1.059, 1.285)	
Metformin				.007		.018		.006
No			1 (Ref.)		1 (Ref.)		1 (Ref.)	
Yes			0.908 (0.846, 0.974)		0.918 (0.855, 0.986)		0.907 (0.845, 0.973)	
Meglitinide				.507		.28		.523
No			1 (Ref.)		1 (Ref.)		1 (Ref.)	
Yes			1.072 (0.873, 1.316)		1.123 (0.910, 1.386)		1.071 (0.868, 1.322)	
Thiazolidinedione				.463		.972		.497
No			1 (Ref.)		1 (Ref.)		1 (Ref.)	
Yes			0.957 (0.850, 1.077)		0.998 (0.880, 1.131)		0.958 (0.845, 1.085)	
DPP-4 inhibitor				.722		.615		.524
No			1 (Ref.)		1 (Ref.)		1 (Ref.)	
Yes			0.981 (0.881, 1.091)		1.031 (0.915, 1.161)		1.039 (0.923, 1.170)	
Alpha -Glucosidase inhibitor				.843		.167		.513
No			1 (Ref.)		1 (Ref.)		1 (Ref.)	
Yes			1.010 (0.918, 1.110)		1.092 (0.964, 1.238)		1.042 (0.921, 1.180)	
Sulfonylurea				.322		.746		.068
No			1 (Ref.)		1 (Ref.)		1 (Ref.)	
Yes			0.964 (0.898, 1.036)		0.988 (0.916, 1.065)		0.933 (0.867, 1.005)	

^a^ Model 1 was adjusted for age, sex, income, BMI, abdominal obesity, smoking, drinking, exercise, hypertension, dyslipidemia, FBG, HDL-C, LDL-C, TG, history of stroke, DM duration, number of OHA.

^b^ Model 2 was adjusted for all variables in Model 1, excluding the number of OHA, and included all antidiabetic medications.

^c^ Model 3 was adjusted for all variables in Model 2, and including number of OHA.

^d^ Model 4 was adjusted for all variables in Model 3, and heart failure, coronary heart disease, COPD.

BMI, body mass index; BP, blood pressure; CI, confidence interval; CKD, chronic kidney disease, COPD, chronic obstructive pulmonary disease; CVD, cardiovascular disease; DM, Diabetes mellitus; DPP-4, dipeptidyl peptidase 4; HDL-C, high density lipoprotein cholesterol; HR, hazard ratio; LDL-C, low density lipoprotein cholesterol; OHA, oral hypoglycemic agent; PY, pack-years; TAAD, thoracic aortic aneurysm and dissection; TG, triglycerides.

Comorbidities (hypertension, dyslipidemia, history of stroke, heart failure, coronary heart disease, and chronic obstructive pulmonary disease), smoking, low HDL-C level, and abdominal obesity were associated with an increased risk of TAAD. Since the HR in Model 2, which excludes OHAs due to concerns about multicollinearity, is not significantly different from the HR in Model 4, which includes OHAs, we have presented the HR from Model 4 as the representative value.

**Figure 2:**
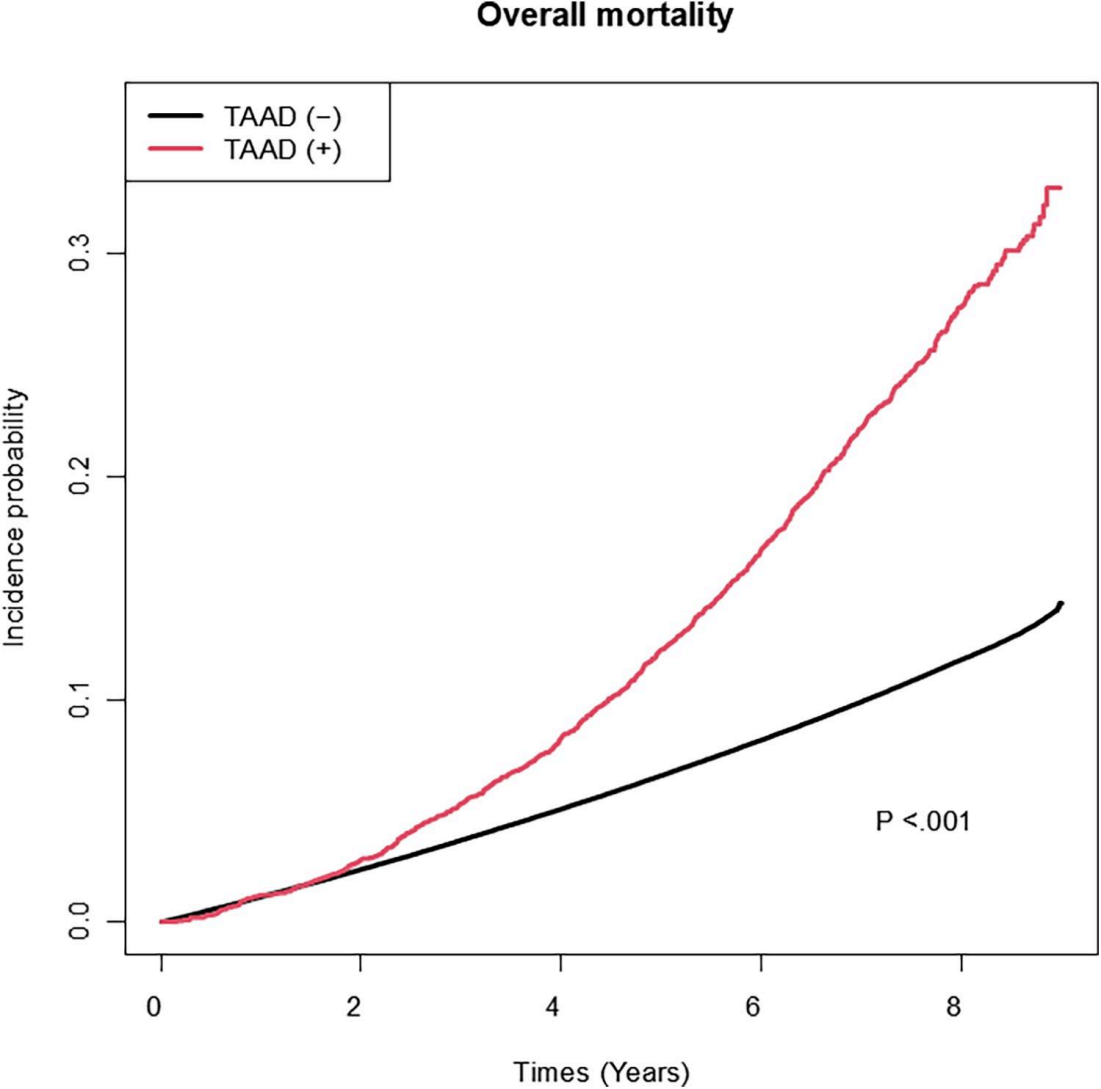
Kaplan–Meier survival curves comparing patients with and without TAAD.

**Figure 3:**
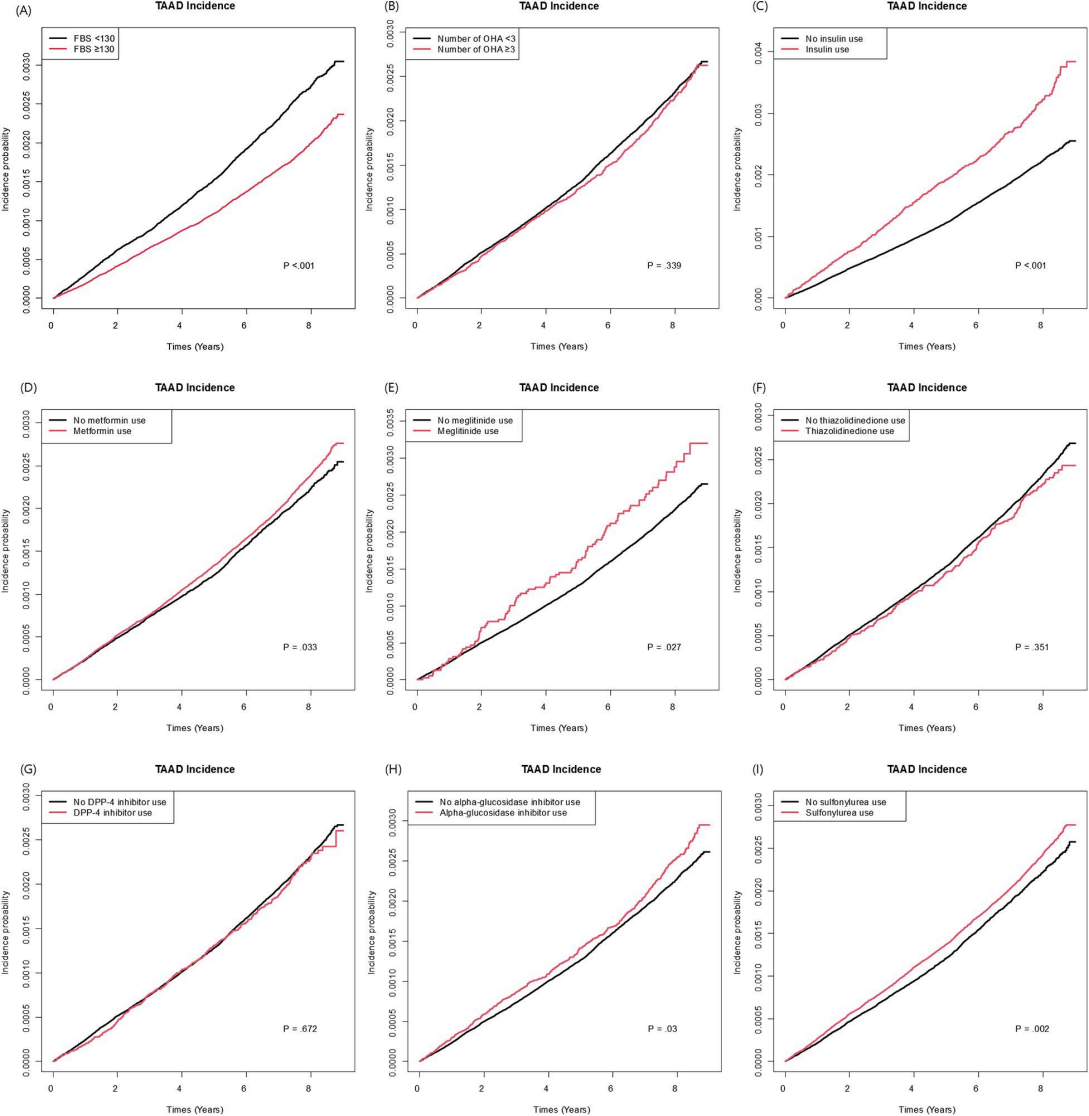
Cumulative incidence of TAAD according to diabetes-specific risk factors: **(A)** fasting blood glucose (<130 vs. ≥130). **(B)** Number of oral hypoglycemic agents (<3 vs. ≥3). **(C)** Insulin use **(D)** Metformin use. **(E)** Meglitinide use. **(F)** Thiazolidinedione. **(G)** DPP-4 inhibitor use. **(H)** Alpha-glucosidase inhibitor use/**(I)** Sulfonylurea use.

Among the variables specific to diabetes, the risk of TAAD was lower in patients with a longer diabetes duration (HR: 0.97, 95% CI: 0.96–0.99), those with FBG level >130 mg/dl (HR: 0.90, 95% CI: 0.84–0.95), and those using metformin (HR: 0.91 95% CI: 0.85–0.97). However, the insulin use (HR: 1.17, 95% CI: 1.06–1.29) was associated with an increased risk of TAAD. Table 3 compares the effect estimates of major cardiovascular risk factors between two previous studies conducted in the general population and the current diabetic cohort. Kaplan–Meier survival analysis revealed that patients diagnosed with TAAD exhibited markedly reduced survival compared to those without TAAD (Figure 2). In addition, we analyzed cumulative incidence of TAAD stratified by major cardiovascular risk factors, including age, sex, abdominal obesity, smoking, alcohol, hypertension, dyslipidemia, HDL-C, heart failure, coronary heart disease, and chronic obstructive pulmonary disease (Supplementary Figures S1–S10, respectively). Furthermore, we examined the cumulative incidence of TAAD according to diabetes-specific factors, including FBG level, number of OHAs, insulin use, and individual antidiabetic medications such as metformin, meglitinide, thiazolidinedione, DPP-4 inhibitors, alpha-glucosidase inhibitors, and sulfonylureas (Figure 3A–I). These analyses revealed differing TAAD incidence patterns across diabetic subgroups, further supporting the associations observed in our multivariable model.

**Table 3:** Comparison of effect estimates for major risk factors for TAAD between general population studies and the current diabetic cohort.

Variables	An et al, (18) 2020		Pham et al, (19) 2024		Current study	
	OR (95% CI)	*P* value	OR (95% CI)	*P* value	OR (95% CI)	*P* value
Age	1.1 (1.1, 1.1)	<.001	2.0 (1.7, 2.4)	<.001	1.1 (1.1, 1.1)	<.001
Sex		<.001		<.001		<.001
Male	3.1 (2.0, 4.9)		2.9 (2.1, 4.1)		1.4 (1.3, 1.5)	
Female	1 (Ref.)		1 (Ref.)		1 (Ref.)	
Hypertension		.075		<.001		<.001
No	1 (Ref.)		1 (Ref.)		1 (Ref.)	
Yes	1.4 (1.0, 2.0)		2.0 (1.5, 2.8)		1.7 (1.5, 1.8)	
Diabetes		<.001		.1	–	–
No	1 (Ref.)		1 (Ref.)		–	
Yes	0.4 (0.2, 0.6)		0.4 (0.1, 1.3)		–	
Dyslipidemia		.04		.6		<.001
No	1 (Ref.)		1 (Ref.)		1 (Ref.)	
Yes	1.5 (1.0, 2.1)		0.9 (0.6–1.4)		1.2 (1.1, 1.3)	
CKD		.005		–		<.001
No	1 (Ref.)		–		1 (Ref.)	
Yes	1.5 (1.0, 1.2)		–		1.3 (1.2, 1.4)	
Smoking		<.001		.86		<.001
No (Ref.)	1 (Ref.)		1 (Ref.)		1 (Ref.)	
Current or former	2.1 (1.4, 3.0)		0.9 (0.7–1.3)		–	
Ex & <20 PY	–		–		1.1 (1.0, 1.3)	
Ex & ≥20 PY	–		–		1.5 (1.4, 1.7)	
Current & <20 PY	–		–		1.6 (1.5, 1.9)	
Current & ≥20 PY	–		–		1.8 (1.7, 2.0)	

### Independent predictors of the development of TAD, TAA, and TAAA

Risk factors of TAD, TAA, and TAAA individually are presented in Supplementary Tables SVI–SVIII, respectively. Age and hypertension were robust and consistent predictors across all three outcomes. Abdominal obesity was significantly associated with risk in TAD (HR: 1.23, 95% CI: 1.10–1.37), TAA (HR: 1.34, 95% CI: 1.22–1.47), and TAAA (HR: 1.29, 95% CI: 1.09–1.53). Male sex was most strongly associated with TAAA (HR: 1.76, 95% CI: 1.47–2.10), followed by TAA (HR: 1.54, 95% CI: 1.41–1.68), and was weakest for TAD (HR: 1.19, 95% CI: 1.10–1.29). FBG > 130 mg/dl was associated with lower risk across TAD (HR: 0.89, 95% CI: 0.84–0.94), TAA (HR: 0.87, 95% CI: 0.82–0.93), and TAAA (HR: 0.88, 95% CI: 0.78–1.00). Similarly, diabetes duration ≥5 years was associated with decreased risk in all three outcomes, although more modestly for TAAA (HR: 0.94, 95% CI: 0.81–1.09). In contrast, insulin use remained a strong independent risk factor for TAD (HR: 1.26, 95% CI: 1.14–1.39) and TAA (HR: 1.25, 95% CI: 1.12–1.40), while it did not reach significance in TAAA (HR: 1.21, 95% CI: 0.95–1.54). Metformin use was protective for TAD (HR: 0.91, 95% CI: 0.85–0.99), but not significantly associated with TAA or TAAA. Interaction analysis between age and sex showed no significant interactions across adjusted models (Supplementary Table SIX).

## Discussion

Several pathophysiological mechanisms with regard to the protective effect of diabetes on TAAD have been explored. In diabetic patients, the glycosaminoglycans in the extracellular matrix of the aorta is decreased, which is thought to increase intramural stress and initiate aortic media delamination (8). In addition, neoangiogenesis in aortic tissue is inhibited in diabetic patients, which may prevent TAAD development (20). Finally, hyperglycemia is associated with decreased inflammatory cells infiltration into the medial layer of the aorta, possibly inhibiting the progression of TAAD (1).

Although a previous study by Hsu et al. reported that antidiabetic agents protected against AAA in diabetic patients (21), TAADs were not investigated. We found that patients with a longer diabetes duration were associated with a decreased risk of TAAD. These patients are likely to have been exposed to prolonged hyperglycemia, suggesting the possibility of being in a more advanced state of the disease. One possible explanation is that prolonged hyperglycemia may lead to structural modifications in the aortic wall, such as increased collagen fibers cross-linking and changes in the extracellular matrix, which can increase arterial stiffness and prevent TAAD formation. Indeed, a previous study reported that more advanced diabetes is significantly associated with a reduced risk of aortic aneurysms (22), consistent with our results.

Among antidiabetic medications, only metformin was linked to a decreased risk of TAAD. It directly inhibits NF-kappaB through the PI3K-Akt pathway, reducing vascular inflammation and AAA growth (23). While a prior study showed metformin use was associated with lower AAA risk (24), its link to TAAD has been less explored. Ma et al. demonstrated that metformin restored contractile phenotype and mitochondrial function in a thoracic aortic tissue model, suggesting its protective role against TAAD (13). However, Vigac et al. found no protective effect of metformin on aortic aneurysms, but their study had limitations, including a small sample size and lack of data on connective tissue disease (14).

While metformin protected against TAAD, insulin therapy had the opposite effect. This may be because insulin counteracts the protective effects of hyperglycemia on elastase-induced AAA formation (20). These findings align with previous reports of a negative association between hyperinsulinemia and AAA diameter (25). Although the exact mechanism is unclear, metformin's effects on inflammation and extracellular matrix remodeling may explain its protective role against aneurysm development (8).

Since this study was conducted on a Korean population, the findings may not be fully generalizable to other ethnic groups. Differences in body composition and responses to medications could influence the outcomes. Notably, the prevalence of aortic aneurysm is generally lower in Asians, though similar rates have been observed among those with cardiovascular risk factors (26). Nevertheless, findings from both Asian and Western studies suggest a potentially shared biological mechanism involving decorin, a small leucine-rich proteoglycan, which may underlie the protective effect of diabetes on aneurysm formation (27, 28). Furthermore, inverse relationships between diabetes and TAAD have been demonstrated in a previous Western study (2). Further research conducted across diverse populations would provide a stronger foundation for drawing conclusions that can be broadly applicable.

2022 ACC/AHA guideline for the diagnosis and management of aortic disease emphasize the importance of smoking cessation in reducing the risk of TAAD (29). Our study revealed a clear dose-dependent relationship between smoking and the risk of TAAD. Compared to non-smokers, ex-smokers with a history of ≥20 pack-years showed a significantly higher risk (HR: 1.53), while current smokers exhibited the greatest risk, particularly those with ≥20 pack-years (HR: 1.83). These results emphasize the cumulative impact of smoking and the importance of cessation in TAAD prevention. The guidelines also stress the management of traditional risk factors, such as hypertension and dyslipidemia, which was similarly reflected in our findings. However, specific recommendations on risk modification in diabetic patients are lacking. Our results suggest that longer diabetes duration may have a protective effect and that metformin use could be a preferable option for those at risk of developing TAAD, offering valuable insights for surveillance. Further exploration of diabetes-specific risk factors could improve risk stratification and management strategies in this population.

Accurately estimating the true incidence of TAAD is essential for understanding the full impact of this condition. Our study recorded all 40 cases of death upon hospital arrival, excluding only pre-hospital deaths. Previous studies report that 17.6% of acute TAD patients (30) and 7% of ruptured aortic aneurysm cases died before reaching the hospital (31). Therefore, it is likely that the potential bias in our results is limited, but it cannot be entirely ruled out.

Several studies conducted in the general population have identified traditional cardiovascular risk factors—such as increased age, male sex, hypertension, CKD, and smoking—as major contributors to the development of aortic aneurysms and dissections, which is consistent with the findings of the current study (4, 18, 19, 32). In our analysis focusing on individuals with diabetes, insulin use was associated with an increased risk of thoracic aortic disease, whereas longer diabetes duration and higher fasting glucose levels were inversely associated with risk. These contrasting patterns suggest that glycemic control and the stage of diabetes progression may play distinct roles in modifying aortic disease risk among diabetic patients, underscoring the need for tailored risk stratification in this population.

### Study limitations

There are several limitations to this study. First, imaging study results could not be obtained, as we used data from the NHIS claims database based on a code system. Therefore, the exact cut-off value for defining aneurysm remains uncertain. However, clinical diagnoses are typically established through CT scans, and it is reasonable to assume that the diagnosis in this context was made using one of the recognized diagnostic criteria. The imaging interpretation would have been conducted by thoracic surgeons, cardiologists, or radiologists, who are trained to review such cases. Second, this database did not include HbA1c results, which serve as a more accurate marker than FBG for assessing the level of diabetes control. Third, we could not conduct detailed analyses on the dosage and different OHAs duration, which limits the ability to assess the dose-response relationship and determine the optimal treatment regimen. Fourth, pre-hospital deaths were not considered, which could have resulted in underestimation of the true incidence of TAAD. Fifth, while the study demonstrates associations, causality cannot be definitively established. Although we included all pertinent variables available in the claims data, the possibility of residual confounding by unmeasured variables may still exist. A prospective study with detailed analyses of medication usage or randomized trials may be beneficial to explore the impact of metformin and other antidiabetic medications. Sixth, our study is observational in nature, and limited in elucidating the mechanism. Further studies are warranted on the association between DM/ antidiabetic medication and aortic pathology and possibly elucidate the underlying biological mechanisms. Finally, the findings of our study may not be generalizable to other racial and ethnic groups. Given the difference in the nature of diabetes mellitus and TAAD between the ethnic groups, further studies including diverse populations would be needed.

## Conclusions

Our study suggests that a longer diabetes duration, FBG level >130 mg/dl and metformin use are associated with a lower risk of TAAD in diabetic patients. These findings highlight the potential protective role of tailored glucose control and treatment strategies against TAAD. However, further research is needed to validate these findings and explore the underlying mechanisms. A collaborative effort among healthcare providers could lead to comprehensive guidelines tailored to individual needs to reduce TAAD incidence and severity in diabetic patients. Until then, vigilant monitoring of TAAD risk factors and personalized care are essential for optimizing patient outcomes.

## Data Availability

The data analyzed in this study is subject to the following licenses/restrictions: The data will be made accessible upon approval of a proposal by the the National Health Insurance Service (NHIS) Database. Requests to access these datasets should be directed to https://nhiss.nhis.or.kr/
