# Supplementary material for: Risk factors for thoracic aortic aneurysm and dissection among diabetic patients: a nationwide population-based study
Source: Front Cardiovasc Med. 2025 Oct 7;12:1569886. doi: 10.3389/fcvm.2025.1569886 (PMC12537662; doi:10.3389/fcvm.2025.1569886)
Supplement: Supplementary file 1 [file Datasheet1.docx]

**Supplementary Table I.** Diagnostic codes for aortic aneurysm and aortic dissection

| **ICD-10 code** | **Diagnosis** |
| --- | --- |
| I71.0 | Dissection of aorta |
| I71.1 | Thoracic aortic aneurysm, ruptured |
| I71.2 | Thoracic aortic aneurysm, without rupture |
| I71.3 | Abdominal aortic aneurysm, ruptured |
| I71.4 | Abdominal aortic aneurysm, without rupture |
| I71.5 | Thoracoabdominal aortic aneurysm, ruptured |
| I71.6 | Thoracoabdominal aortic aneurysm, without rupture |
| I71.8 | Aortic aneurysm of unspecified site, ruptured |
| I71.9 | Aortic aneurysm of unspecified site, without rupture |
| I71.00 | Dissection of aorta, unspecified |
| I71.01 | Dissection of aorta, thoracic |

*ICD*, international classification of diseases.

**Table II.** Baseline characteristics of patients with and without thoracic aortic dissection

|  | **TAD** | | |
| --- | --- | --- | --- |
|  | **No (n = 2,325,959)** | **Yes (n = 2,388)** | ***P* value** |
| Age |  |  | <.001 |
| <65 years | 1,566,710 (67.4) | 1,078 (45.1) |  |
| ≥65 years | 759,249 (32.6) | 1,310 (54.9) |  |
| Female | 979,080 (42.1) | 976 (40.9) | 0.227 |
| Income, lowest quartile | 495,135 (21.3) | 554 (23.2) | 0.023 |
| Hypertension | 1,379,123 (59.3) | 1,804 (75.5) | <.001 |
| Dyslipidemia | 1,003,717 (43.2) | 1,208 (50.6) | <.001 |
| CKD | 284,667 (12.2) | 517 (21.7) | <.001 |
| History of stroke | 48,568 (2.1) | 118 (4.9) | <.001 |
| Smoking |  |  | <.001 |
| Never | 1,346,188 (57.9) | 1,286 (53.9) |  |
| Ex & <20 PY | 215,581 (9.3) | 180 (7.5) |  |
| Ex & ≥20 PY | 215,993 (9.3) | 301 (12.6) |  |
| Current & <20 PY | 207,616 (8.9) | 205 (8.6) |  |
| Current & ≥20 PY | 340,581 (14.6) | 416 (17.4) |  |
| Drinking |  |  | <.001 |
| Non | 1,386,103 (59.6) | 1,584 (66.3) |  |
| Mild | 718,080 (30.9) | 616 (25.8) |  |
| Heavy | 221,776 (9.5) | 188 (7.9) |  |
| Regular exercise | 489,993 (21.1) | 485 (20.3) | 0.365 |
| BMI, kg/m2 |  |  | 0.08 |
| <18.5 | 35,404 (1.5) | 33 (1.4) |  |
| <23 | 581,437 (25.0) | 568 (23.8) |  |
| <25 | 588,756 (25.3) | 576 (24.1) |  |
| <30 | 956,865 (41.1) | 1,017 (42.6) |  |
| ≥30 | 163,497 (7.0) | 194 (8.1) |  |
| Abdominal obesity | 896,678 (38.6) | 1,126 (47.2) | <.001 |
| DM duration, ≥5 years | 765,550 (32.9) | 828 (34.7) | 0.067 |
| OHA, ≥3 | 361,265 (15.5) | 350 (14.7) | 0.238 |
| Insulin | 230,175 (9.9) | 312 (13.1) | <.001 |
| Metformin | 1,180,689 (50.8) | 1,205 (50.5) | 0.769 |
| Meglitinide | 42,984 (1.9) | 58 (2.4) | 0.035 |
| Thiazolidinedione | 159,375 (6.9) | 156 (6.5) | 0.537 |
| DPP-4 inhibitor | 215,943 (9.3) | 199 (8.3) | 0.11 |
| Alpha-glucosidase inhibitor | 282,856 (12.2) | 305 (12.8) | 0.361 |
| Sulfonylurea | 1,026,392 (44.1) | 1,108 (46.4) | 0.026 |
| Systolic BP, mmHg | 129.3±15.9 | 131.4±16.8 | <.001 |
| Fasting glucose, mg/dL | 143.8±46.4 | 136.5±44.2 | <.001 |
| Total cholesterol, mg/dL | 196.1±42.5 | 191.9±42.8 | <.001 |
| HDL-C, mg/dL | 51.9±22.2 | 51.1±29.4 | 0.085 |
| LDL-C, mg/dL | 111.3±40.8 | 109.3±44.1 | 0.014 |

Values are presented as the as the mean ± standard deviation or geometric mean [95% confidence interval] for continuous data and as number (%) for categorical data.

Abbreviations: BMI, body mass index; BP, blood pressure; CKD, chronic kidney disease; CVD, cardiovascular disease; DM, diabetes mellitus; DPP-4 inhibitor, dipeptidyl peptidase 4 inhibitors; GFR, glomerular filtration rate; HDL-C, high density lipoprotein cholesterol; LDL-C, low density lipoprotein cholesterol; OHA, oral hypoglycemic agent; PY, pack-years; TAD, thoracic aortic dissection.

**Table III.** Baseline characteristics of patients with and without thoracic aortic aneurysm

|  | **TAA** | | |
| --- | --- | --- | --- |
|  | **No (n = 2,326,663)** | **Yes (n = 1,684)** | ***P* value** |
| Age |  |  | <.001 |
| <65 years | 1,567,111 (67.4) | 677 (40.2) |  |
| ≥65 years | 759,552 (32.6) | 1,007 (59.8) |  |
| Female | 979,442 (42.1) | 614 (36.5) | <.001 |
| Income, lowest quartile | 495,363 (21.3) | 326 (19.4) | 0.053 |
| Hypertension | 1,379,582 (59.3) | 1,345 (79.9) | <.001 |
| Dyslipidemia | 1,004,037 (43.2) | 888 (52.7) | <.001 |
| CKD | 284,783 (12.2) | 401 (23.8) | <.001 |
| History of stroke | 48,593 (2.1) | 93 (5.5) | <.001 |
| Smoking |  |  | <.001 |
| Never | 1,346,575 (57.9) | 899 (53.4) |  |
| Ex & <20 PY | 215,628 (9.3) | 133 (7.9) |  |
| Ex & ≥20 PY | 216,043 (9.3) | 251 (14.9) |  |
| Current & <20 PY | 207,693 (8.9) | 128 (7.6) |  |
| Current & ≥20 PY | 340,724 (14.6) | 273 (16.2) |  |
| Drinking |  |  | <.001 |
| Non | 1,386,615 (59.6) | 1,072 (63.7) |  |
| Mild | 718,223 (30.9) | 473 (28.1) |  |
| Heavy | 221,825 (9.5) | 139 (8.2) |  |
| Regular exercise | 490,137 (21.1) | 341 (20.3) | 0.411 |
| BMI, kg/m2 |  |  | 0.1 |
| <18.5 | 35,416 (1.5) | 21 (1.3) |  |
| <23 | 581,592 (25.0) | 413 (24.5) |  |
| <25 | 588,938 (25.3) | 394 (23.4) |  |
| <30 | 957,165 (41.1) | 717 (42.6) |  |
| ≥30 | 163,552 (7.0) | 139 (8.2) |  |
| Abdominal obesity | 896,977 (38.6) | 827 (49.1) | <.001 |
| DM duration, ≥5 years | 765,794 (32.9) | 584 (34.7) | 0.123 |
| OHA, ≥3 | 361,364 (15.5) | 251 (14.9) | 0.478 |
| Insulin | 230,259 (9.9) | 228 (13.5) | <.001 |
| Metformin | 1,180,991 (50.8) | 903 (53.6) | 0.019 |
| Meglitinide | 43,005 (1.9) | 37 (2.4) | 0.288 |
| Thiazolidinedione | 159,406 (6.9) | 125 (7.4) | 0.353 |
| DPP-4 inhibitor | 215,986 (9.3) | 156 (9.3) | 0.978 |
| Alpha-glucosidase inhibitor | 282,933 (12.2) | 228 (13.5) | 0.084 |
| Sulfonylurea | 1,026,671 (44.1) | 829 (49.2) | <.001 |
| Systolic BP, mmHg | 129.3±15.9 | 131.7±16.9 | <.001 |
| Fasting glucose, mg/dL | 143.8±46.4 | 136.5±44.4 | <.001 |
| Total cholesterol, mg/dL | 196.1±42.5 | 189.8±45.1 | <.001 |
| HDL-C, mg/dL | 51.9±22.2 | 49.6±16.5 | <.001 |
| LDL-C, mg/dL | 111.3±40.9 | 108.0±40.4 | 0.001 |

Values are presented as the as the mean ± standard deviation or geometric mean [95% confidence interval] for continuous data and as number (%) for categorical data.

Abbreviations: BMI, body mass index; BP, blood pressure; CKD, chronic kidney disease; CVD, cardiovascular disease; DM, diabetes mellitus; DPP-4 inhibitor, dipeptidyl peptidase 4 inhibitors; GFR, glomerular filtration rate; HDL-C, high density lipoprotein cholesterol; LDL-C, low density lipoprotein cholesterol; OHA, oral hypoglycemic agent; PY, pack-years; TAA, thoracic aortic aneurysm.

**Table IV.** Baseline characteristics of patients with and without thoracoabdominal aortic aneurysm

|  | **TAAA** | | |
| --- | --- | --- | --- |
|  | **No (n = 2,327,576)** | **Yes (n = 771)** | ***P* value** |
| Age |  |  | <.0001 |
| <65 years | 1,567,474 (67.3) | 314 (40.7) |  |
| ≥65 years | 760,102 (32.7) | 457 (59.3) |  |
| Female | 979,808 (42.1) | 248 (32.2) | <.001 |
| Income, lowest quartile | 495,506 (21.3) | 183 (23.7) | 0.097 |
| Hypertension | 1,380,357 (59.3) | 570 (73.9) | <.0001 |
| Dyslipidemia | 1,004,554 (43.2) | 371 (48.1) | 0.005 |
| CKD | 285,019 (12.2) | 165 (21.4) | <.0001 |
| History of stroke | 48,643 (2.1) | 43 (5.6) | <.0001 |
| Smoking |  |  | <.001 |
| Never | 1,347,110 (57.9) | 364 (47.2) |  |
| Ex & <20 PY | 215,690 (9.3) | 71 (9.2) |  |
| Ex & ≥20 PY | 216,186 (9.3) | 108 (14.0) |  |
| Current & <20 PY | 207,749 (8.9) | 72 (9.3) |  |
| Current & ≥20 PY | 340,841 (14.6) | 156 (20.2) |  |
| Drinking |  |  | <.001 |
| Non | 1,387,199 (59.6) | 488 (63.3) |  |
| Mild | 718,465 (30.9) | 231 (30.0) |  |
| Heavy | 221,912 (9.5) | 52 (6.7) |  |
| Regular exercise | 490,324 (21.1) | 154 (20.0) | 0.457 |
| BMI, kg/m2 |  |  | 0.788 |
| <18.5 | 35,427 (1.5) | 10 (1.3) |  |
| <23 | 581,822 (25.0) | 183 (23.7) |  |
| <25 | 589,138 (25.3) | 194 (25.2) |  |
| <30 | 957,559 (41.1) | 323 (41.9) |  |
| ≥30 | 163,630 (7.0) | 61 (7.9) |  |
| Abdominal obesity | 897,438 (38.6) | 366 (47.5) | <.0001 |
| DM duration, ≥5 years | 766,113 (32.9) | 265 (34.4) | 0.39 |
| OHA, ≥3 | 361,487 (15.5) | 128 (16.6) | 0.412 |
| Insulin | 230,393 (9.9) | 94 (12.2) | 0.033 |
| Metformin | 1,181,491 (50.8) | 403 (52.3) | 0.402 |
| Meglitinide | 43,025 (1.9) | 17 (2.2) | 0.463 |
| Thiazolidinedione | 159,476 (6.9) | 55 (7.1) | 0.757 |
| DPP-4 inhibitor | 216,073 (9.3) | 69 (9.0) | 0.75 |
| Alpha-glucosidase inhibitor | 283,039 (12.2) | 122 (15.8) | 0.002 |
| Sulfonylurea | 1,027,123 (44.1) | 377 (48.9) | 0.008 |
| Systolic BP, mmHg | 129.3±15.9 | 131.6±17.4 | <.0001 |
| Fasting glucose, mg/dL | 143.8±46.4 | 134.5±40.0 | <.0001 |
| Total cholesterol, mg/dL | 196.1±42.5 | 193.0±42.9 | 0.027 |
| HDL-C, mg/dL | 51.9±22.2 | 49.6±14.6 | 0.004 |
| LDL-C, mg/dL | 111.3±40.8 | 111.7±45.4 | 0.812 |

Values are presented as the as the mean ± standard deviation or geometric mean [95% confidence interval] for continuous data and as number (%) for categorical data.

Abbreviations: BMI, body mass index; BP, blood pressure; CKD, chronic kidney disease; CVD, cardiovascular disease; DM, diabetes mellitus; DPP-4 inhibitor, dipeptidyl peptidase 4 inhibitors; GFR, glomerular filtration rate; HDL-C, high density lipoprotein cholesterol; LDL-C, low density lipoprotein cholesterol; OHA, oral hypoglycemic agent; PY, pack-years; TAAA, thoracoabdominal aortic aneurysm.

**Supplementary Table V.** Multivariate analysis including all variables for development of TAAD

|  | **Model 1^a^** | ***P* value** | **Model 2 ^b^** | ***P* value** | **Model 3^c^** | ***P* value** | **Model 4^d^** | ***P* value** |
| --- | --- | --- | --- | --- | --- | --- | --- | --- |
| Age, year | 1.057 (1.053, 1.061) | <.001 | 1.057 (1.053, 1.061) | <.001 | 1.057 (1.053, 1.061) | <.001 | 1.054 (1.051, 1.058) | <.001 |
| Sex |  | <.001 |  | <.001 |  | <.001 |  | <.001 |
| Male | 1.391 (1.279, 1.512) |  | 1.385 (1.273, 1.506) |  | 1.385 (1.274, 1.507) |  | 1.369 (1.259, 1.489) |  |
| Female | 1 (Ref.) |  | 1 (Ref.) |  | 1 (Ref.) |  | 1 (Ref.) |  |
| Income |  | .037 |  | .038 |  | .037 |  | .022 |
| Q2-4 | 1 (Ref.) |  | 1 (Ref.) |  | 1 (Ref.) |  | 1 (Ref.) |  |
| Q1 | 1.079 (1.005, 1.158) |  | 1.078 (1.004, 1.157) |  | 1.078 (1.005, 1.158) |  | 1.087 (1.012, 1.166) |  |
| BMI, kg/m2 |  | .004 |  | .004 |  | .004 |  | .003 |
| <18.5 | 0.913 (0.700, 1.190) |  | 0.908 (0.697, 1.184) |  | 0.909 (0.697, 1.185) |  | 0.922 (0.707, 1.202) |  |
| <23 | 1 (Ref.) |  | 1 (Ref.) |  | 1 (Ref.) |  | 1 (Ref.) |  |
| <25 | 0.898 (0.823, 0.979) |  | 0.900 (0.825, 0.982) |  | 0.899 (0.824, 0.981) |  | 0.891 (0.817, 0.972) |  |
| <30 | 0.923 (0.843, 1.010) |  | 0.926 (0.846, 1.014) |  | 0.925 (0.845, 1.013) |  | 0.913 (0.834, 0.999) |  |
| ≥30 | 1.099 (0.956, 1.262) |  | 1.105 (0.962, 1.270) |  | 1.103 (0.960, 1.268) |  | 1.083 (0.943, 1.244) |  |
| Abdominal obesity |  | <.001 |  | <.001 |  | <.001 |  | <.001 |
| No | 1 (Ref.) |  | 1 (Ref.) |  | 1 (Ref.) |  | 1 (Ref.) |  |
| Yes | 1.252 (1.162, 1.349) |  | 1.253 (1.163, 1.350) |  | 1.253 (1.163, 1.350) |  | 1.243 (1.154, 1.340) |  |
| Smoking |  | <.001 |  | <.001 |  | <.001 |  | <.001 |
| Non | 1 (Ref.) |  | 1 (Ref.) |  | 1 (Ref.) |  | 1 (Ref.) |  |
| Ex & <20 PY | 1.121 (0.992, 1.265) |  | 1.121 (0.993, 1.266) |  | 1.121 (0.993, 1.266) |  | 1.121 (0.993, 1.266) |  |
| Ex & ≥20 PY | 1.526 (1.375, 1.693) |  | 1.530 (1.379, 1.698) |  | 1.529 (1.378, 1.697) |  | 1.509 (1.360, 1.675) |  |
| Current & <20 PY | 1.625 (1.440, 1.833) |  | 1.624 (1.440, 1.832) |  | 1.625 (1.440, 1.833) |  | 1.643 (1.457, 1.854) |  |
| Current & ≥20 PY | 1.828 (1.656, 2.019) |  | 1.834 (1.660, 2.025) |  | 1.833 (1.660, 2.025) |  | 1.838 (1.665, 2.030) |  |
| Drinking |  | .002 |  | .001 |  | .002 |  | 0.007 |
| Non | 1 (Ref.) |  | 1 (Ref.) |  | 1 (Ref.) |  | 1 (Ref.) |  |
| Mild | 0.897 (0.830, 0.969) |  | 0.894 (0.828, 0.966) |  | 0.895 (0.828, 0.967) |  | 0.907 (0.840, 0.980) |  |
| Heavy | 0.828 (0.733, 0.935) |  | 0.825 (0.730, 0.932) |  | 0.826 (0.731, 0.933) |  | 0.848 (0.750, 0.958) |  |
| Regular exercise |  | .114 |  | .120 |  | .116 |  | 0.1 |
| No | 1 (Ref.) |  | 1 (Ref.) |  | 1 (Ref.) |  | 1 (Ref.) |  |
| Yes | 0.942 (0.875, 1.014) |  | 0.943 (0.876, 1.015) |  | 0.943 (0.876, 1.015) |  | 0.939 (0.872, 1.011) |  |
| Hypertension |  | <.001 |  | <.001 |  | <.001 |  | <.001 |
| No | 1 (Ref.) |  | 1 (Ref.) |  | 1 (Ref.) |  | 1 (Ref.) |  |
| Yes | 1.726 (1.597, 1.865) |  | 1.733 (1.604, 1.873) |  | 1.731 (1.602, 1.871) |  | 1.639 (1.516, 1.772) |  |
| Dyslipidemia |  | <.001 |  | <.001 |  | <.001 |  | <.001 |
| No | 1 (Ref.) |  | 1 (Ref.) |  | 1 (Ref.) |  | 1 (Ref.) |  |
| Yes | 1.294 (1.214, 1.379) |  | 1.305 (1.224, 1.391) |  | 1.302 (1.222, 1.388) |  | 1.223 (1.146, 1.304) |  |
| Heart failure |  |  |  |  |  |  |  | .003 |
| No |  |  |  |  |  |  | 1 (Ref.) |  |
| Yes |  |  |  |  |  |  | 1.252 (1.079, 1.452) |  |
| Coronary heart disease |  |  |  |  |  |  |  | <.001 |
| No |  |  |  |  |  |  | 1 (Ref.) |  |
| Yes |  |  |  |  |  |  | 1.837 (1.712, 1.972) |  |
| COPD |  |  |  |  |  |  |  | .002 |
| No |  |  |  |  |  |  | 1 (Ref.) |  |
| Yes |  |  |  |  |  |  | 1.146 (1.052, 1.249) |  |
| HDL-C, quartile (mg/dL) |  | <.001 |  | <.001 |  | <.001 |  | <.001 |
| Q1 (<41) | 1.239 (1.135, 1.352) |  | 1.243 (1.139, 1.357) |  | 1.242 (1.138, 1.356) |  | 1.215 (1.113, 1.326) |  |
| Q2 (42-49) | 1.054 (0.966, 1.149) |  | 1.057 (0.970, 1.153) |  | 1.056 (0.969, 1.152) |  | 1.041 (0.955, 1.135) |  |
| Q3 (50-58) | 1.004 (0.920, 1.096) |  | 1.006 (0.922, 1.099) |  | 1.006 (0.921, 1.098) |  | 0.998 (0.914, 1.089) |  |
| Q4 (≥59) | 1 (Ref.) |  | 1 (Ref.) |  | 1 (Ref.) |  | 1 (Ref.) |  |
| LDL-C, quartile (mg/dL) |  | .701 |  | .612 |  | .632 |  | 0.889 |
| Q1 (<84) | 1 (Ref.) |  | 1 (Ref.) |  | 1 (Ref.) |  | 1 (Ref.) |  |
| Q2 (85-109) | 0.991 (0.914, 1.075) |  | 0.991 (0.913, 1.075) |  | 0.991 (0.913, 1.075) |  | 1.016 (0.936, 1.102) |  |
| Q3 (110-135) | 0.996 (0.915, 1.084) |  | 0.995 (0.913, 1.083) |  | 0.995 (0.914, 1.083) |  | 1.035 (0.950, 1.127) |  |
| Q4 (≥136) | 0.954 (0.876, 1.038) |  | 0.948 (0.870, 1.032) |  | 0.949 (0.872, 1.033) |  | 1.022 (0.938, 1.113) |  |
| TG, quartile (mg/dL) |  | .016 |  | .015 |  | .016 |  | 0.1 |
| Q1 (<98) | 1 (Ref.) |  | 1 (Ref.) |  | 1 (Ref.) |  | 1 (Ref.) |  |
| Q2 (99-141) | 1.020 (0.939, 1.108) |  | 1.021 (0.939, 1.109) |  | 1.021 (0.940, 1.109) |  | 1.030 (0.948, 1.119) |  |
| Q3 (142-206) | 0.948 (0.870, 1.033) |  | 0.949 (0.871, 1.034) |  | 0.950 (0.872, 1.035) |  | 0.966 (0.887, 1.053) |  |
| Q4 (≥207) | 0.894 (0.816, 0.979) |  | 0.893 (0.815, 0.978) |  | 0.894 (0.816, 0.980) |  | 0.926 (0.845, 1.014) |  |
| FBG, mg/dL |  | <.001 |  | <.001 |  | <.001 |  | <.001 |
| ≤130 | 1 (Ref.) |  | 1 (Ref.) |  | 1 (Ref.) |  | 1 (Ref.) |  |
| >130 | 0.897 (0.845, 0.952) |  | 0.882 (0.830, 0.937) |  | 0.887 (0.835, 0.943) |  | 0.897 (0.844, 0.953) |  |
| CKD |  | <.001 |  | <.001 |  | <.001 |  | <.001 |
| No | 1 (Ref.) |  | 1 (Ref.) |  | 1 (Ref.) |  | 1 (Ref.) |  |
| Yes | 1.391 (1.291, 1.498) |  | 1.379 (1.280, 1.486) |  | 1.379 (1.280, 1.486) |  | 1.339 (1.243, 1.442) |  |
| History of stroke |  | <.001 |  | <.001 |  | <.001 |  | <.001 |
| No | 1 (Ref.) |  | 1 (Ref.) |  | 1 (Ref.) |  | 1 (Ref.) |  |
| Yes | 1.659 (1.447, 1.901) |  | 1.656 (1.445, 1.898) |  | 1.656 (1.446, 1.898) |  | 1.391 (1.163, 1.665) |  |
| Number of OHAs ≥3 |  | .0212 |  |  |  | .063 |  | .165 |
| <3 | 1 (Ref.) |  |  |  | 1 (Ref.) |  | 1 (Ref.) |  |
| ≥3 | 0.903 (0.827, 0.985) |  |  |  | 0.877 (0.764, 1.007) |  | 0.907 (0.791, 1.041) |  |
| DM duration, year | 0.968 (0.958, 0.978) | <.001 | 0.973 (0.962, 0.985) | <.001 | 0.972 (0.960, 0.984) | <.001 | 0.974 (0.963, 0.985) | <.001 |
| Insulin |  | <.001 |  | <.001 |  | <.001 |  | 0.002 |
| No | 1 (Ref.) |  | 1 (Ref.) |  | 1 (Ref.) |  | 1 (Ref.) |  |
| Yes | 1.248 (1.134, 1.374) |  | 1.232 (1.118, 1.358) |  | 1.234 (1.120, 1.360) |  | 1.166 (1.059, 1.285) |  |
| Metformin |  |  |  | .007 |  | .018 |  | .006 |
| No |  |  | 1 (Ref.) |  | 1 (Ref.) |  | 1 (Ref.) |  |
| Yes |  |  | 0.908 (0.846, 0.974) |  | 0.918 (0.855, 0.986) |  | 0.907 (0.845, 0.973) |  |
| Meglitinide |  |  |  | .507 |  | .28 |  | .523 |
| No |  |  | 1 (Ref.) |  | 1 (Ref.) |  | 1 (Ref.) |  |
| Yes |  |  | 1.072 (0.873, 1.316) |  | 1.123 (0.910, 1.386) |  | 1.071 (0.868, 1.322) |  |
| Thiazolidinedione |  |  |  | .463 |  | .972 |  | .497 |
| No |  |  | 1 (Ref.) |  | 1 (Ref.) |  | 1 (Ref.) |  |
| Yes |  |  | 0.957 (0.850, 1.077) |  | 0.998 (0.880, 1.131) |  | 0.958 (0.845, 1.085) |  |
| DPP-4 inhibitor |  |  |  | .722 |  | .615 |  | .524 |
| No |  |  | 1 (Ref.) |  | 1 (Ref.) |  | 1 (Ref.) |  |
| Yes |  |  | 0.981 (0.881, 1.091) |  | 1.031 (0.915, 1.161) |  | 1.039 (0.923, 1.170) |  |
| Alpha -Glucosidase inhibitor |  |  |  | .843 |  | .167 |  | .513 |
| No |  |  | 1 (Ref.) |  | 1 (Ref.) |  | 1 (Ref.) |  |
| Yes |  |  | 1.010 (0.918, 1.110) |  | 1.092 (0.964, 1.238) |  | 1.042 (0.921, 1.180) |  |
| Sulfonylurea |  |  |  | .322 |  | .746 |  | .068 |
| No |  |  | 1 (Ref.) |  | 1 (Ref.) |  | 1 (Ref.) |  |
| Yes |  |  | 0.964 (0.898, 1.036) |  | 0.988 (0.916, 1.065) |  | 0.933 (0.867, 1.005) |  |

*BMI*, body mass index; *CI*, confidence interval; *CKD,* chronic kidney disease; *COPD*, chronic obstructive pulmonary disease; *DM,* Diabetes mellitus; *DPP-4*, inhibitor dipeptidyl peptidase 4 inhibitors; *FBG*, fasting blood glucose; *HDL-C*, high density lipoprotein cholesterol; *HR*, hazard ratio; *LDL-C*, low density lipoprotein cholesterol; *OHA*, oral hypoglycemic agent; *PY*, pack-years; *TAAD*, thoracic aortic aneurysm and dissection; *TG*, triglycerides.

^a^Model 1 was adjusted for age, sex, income, BMI, abdominal obesity, smoking, drinking, exercise, hypertension, dyslipidemia, FBG, HDL-C, LDL-C, TG, CKD, history of stroke, DM duration, number of OHA.

^b^Model 2 was adjusted for all variables in Model 1, excluding the number of OHA, and adding Metformin, Meglitinide, TZD, DPP-4, alpha-glucosidase inhibitor, and Sulfonylurea.

^c^Model 3 was adjusted for all variables in Model 2, and adding number of OHA.

^d^Model 4 was adjusted for all variables in Model 3, and heart failure, coronary heart disease, chronic obstructive pulmonary disease.

**Supplementary Table VI.** Risk of thoracic aortic dissection according to specified risk factors

|  | **Model 1^a^** | ***P* value** | **Model 2 ^b^** | ***P* value** | **Model 3^c^** | ***P* value** |
| --- | --- | --- | --- | --- | --- | --- |
| Age, year | 1.051 (1.046, 1.056) | <.001 | 1.051 (1.046, 1.056) | <.001 | 1.051 (1.046, 1.056) | <.001 |
| Sex |  | .003 |  | .004 |  | .005 |
| Male | 1.191 (1.060, 1.338) |  | 1.182 (1.052, 1.329) |  | 1.183 (1.052, 1.329) |  |
| Female | 1 (Ref.) |  | 1 (Ref.) |  | 1 (Ref.) |  |
| Income |  | .004 |  | .004 |  | .004 |
| Q2-4 | 1 (Ref.) |  | 1 (Ref.) |  | 1 (Ref.) |  |
| Q1 | 1.151 (1.047, 1.267) |  | 1.151 (1.046, 1.266) |  | 1.151 (1.046, 1.266) |  |
| BMI, kg/m2 |  | .285 |  | .282 |  | .282 |
| <18.5 | 0.995 (0.700, 1.416) |  | 0.988 (0.694, 1.405) |  | 0.988 (0.694, 1.405) |  |
| <23 | 1 (Ref.) |  | 1 (Ref.) |  | 1 (Ref.) |  |
| <25 | 0.935 (0.830, 1.053) |  | 0.938 (0.833, 1.056) |  | 0.938 (0.833, 1.056) |  |
| <30 | 0.925 (0.817, 1.047) |  | 0.929 (0.820, 1.051) |  | 0.928 (0.820, 1.051) |  |
| ≥30 | 1.071 (0.885, 1.296) |  | 1.081 (0.892, 1.308) |  | 1.080 (0.892, 1.308) |  |
| Abdominal obesity |  | <.001 |  | <.001 |  | <.001 |
| No | 1 (Ref.) |  | 1 (Ref.) |  | 1 (Ref.) |  |
| Yes | 1.225 (1.106, 1.358) |  | 1.229 (1.109, 1.362) |  | 1.229 (1.109, 1.362) |  |
| Smoking |  | <.001 |  | <.001 |  | <.001 |
| Non | 1 (Ref.) |  | 1 (Ref.) |  | 1 (Ref.) |  |
| Ex & <20 PY | 1.162 (0.979, 1.379) |  | 1.161 (0.978, 1.378) |  | 1.161 (0.978, 1.378) |  |
| Ex & ≥20 PY | 1.565 (1.350, 1.816) |  | 1.574 (1.357, 1.826) |  | 1.574 (1.357, 1.826) |  |
| Current & <20 PY | 1.784 (1.515, 2.100) |  | 1.784 (1.515, 2.100) |  | 1.784 (1.515, 2.100) |  |
| Current & ≥20 PY | 1.982 (1.727, 2.274) |  | 1.994 (1.738, 2.289) |  | 1.994 (1.738, 2.289) |  |
| Drinking |  | <.001 |  | <.001 |  | <.001 |
| Non | 1 (Ref.) |  | 1 (Ref.) |  | 1 (Ref.) |  |
| Mild | 0.813 (0.730, 0.906) |  | 0.808 (0.725, 0.901) |  | 0.808 (0.725, 0.901) |  |
| Heavy | 0.780 (0.660, 0.923) |  | 0.775 (0.655, 0.917) |  | 0.775 (0.655, 0.917) |  |
| Regular exercise |  | .552 |  | .559 |  | .558 |
| No | 1 (Ref.) |  | 1 (Ref.) |  | 1 (Ref.) |  |
| Yes | 0.970 (0.877, 1.073) |  | 0.970 (0.877, 1.074) |  | 0.970 (0.877, 1.073) |  |
| Hypertension |  | <.001 |  | <.001 |  | <.001 |
| No | 1 (Ref.) |  | 1 (Ref.) |  | 1 (Ref.) |  |
| Yes | 1.685 (1.517, 1.871) |  | 1.696 (1.527, 1.884) |  | 1.696 (1.527, 1.884) |  |
| Dyslipidemia |  | <.001 |  | <.001 |  | <.001 |
| No | 1 (Ref.) |  | 1 (Ref.) |  | 1 (Ref.) |  |
| Yes | 1.246 (1.142, 1.360) |  | 1.265 (1.159, 1.382) |  | 1.265 (1.158, 1.382) |  |
| HDL-C, quartile (mg/dL) |  | <.001 |  | <.001 |  | <.001 |
| Q1 (<41) | 1.182 (1.050, 1.331) |  | 1.189 (1.056, 1.339) |  | 1.189 (1.056, 1.339) |  |
| Q2 (42-49) | 0.957 (0.850, 1.077) |  | 0.962 (0.855, 1.083) |  | 0.962 (0.855, 1.083) |  |
| Q3 (50-58) | 0.914 (0.811, 1.031) |  | 0.917 (0.813, 1.034) |  | 0.917 (0.813, 1.034) |  |
| Q4 (≥59) | 1 (Ref.) |  | 1 (Ref.) |  | 1 (Ref.) |  |
| LDL-C, quartile (mg/dL) |  | .719 |  | .621 |  | .624 |
| Q1 (<84) | 1 (Ref.) |  | 1 (Ref.) |  | 1 (Ref.) |  |
| Q2 (85-109) | 0.993 (0.888, 1.111) |  | 0.993 (0.888, 1.110) |  | 0.993 (0.888, 1.110) |  |
| Q3 (110-135) | 0.966 (0.859, 1.086) |  | 0.964 (0.858, 1.085) |  | 0.965 (0.858, 1.085) |  |
| Q4 (≥136) | 0.940 (0.836, 1.056) |  | 0.930 (0.828, 1.046) |  | 0.930 (0.828, 1.046) |  |
| TG, quartile (mg/dL) |  | .718 |  | .695 |  | .696 |
| Q1 (<98) | 1 (Ref.) |  | 1 (Ref.) |  | 1 (Ref.) |  |
| Q2 (99-141) | 1.051 (0.936, 1.180) |  | 1.053 (0.938, 1.182) |  | 1.053 (0.938, 1.182) |  |
| Q3 (142-206) | 1.020 (0.906, 1.149) |  | 1.022 (0.908, 1.152) |  | 1.023 (0.908, 1.152) |  |
| Q4 (≥207) | 0.986 (0.870, 1.118) |  | 0.986 (0.870, 1.118) |  | 0.986 (0.870, 1.118) |  |
| FBG, mg/dL |  | .043 |  | .009 |  | .010 |
| ≤130 | 1 (Ref.) |  | 1 (Ref.) |  | 1 (Ref.) |  |
| >130 | 0.918 (0.846, 0.997) |  | 0.895 (0.824, 0.973) |  | 0.896 (0.824, 0.974) |  |
| CKD |  | <.001 |  | <.001 |  | <.001 |
| No | 1 (Ref.) |  | 1 (Ref.) |  | 1 (Ref.) |  |
| Yes | 1.401 (1.264, 1.552) |  | 1.381 (1.246, 1.530) |  | 1.381 (1.246, 1.530) |  |
| History of stroke |  | <.001 |  | <.001 |  | <.001 |
| No | 1 (Ref.) |  | 1 (Ref.) |  | 1 (Ref.) |  |
| Yes | 1.659 (1.372, 2.006) |  | 1.656 (1.369, 2.002) |  | 1.656 (1.369, 2.002) |  |
| Number of OHAs ≥3 |  | .063 |  |  |  | .811 |
| <3 | 1 (Ref.) |  |  |  | 1 (Ref.) |  |
| ≥3 | 0.892 (0.790, 1.006) |  |  |  | 0.977 (0.806, 1.184) |  |
| DM duration, year | 0.963 (0.950, 0.977) | <.001 | 0.976 (0.960, 0.992) | .003 | 0.975 (0.959, 0.992) | .003 |
| Insulin |  | <.001 |  | <.001 |  | <.001 |
| No | 1 (Ref.) |  | 1 (Ref.) |  | 1 (Ref.) |  |
| Yes | 1.286 (1.127, 1.467) |  | 1.256 (1.099, 1.436) |  | 1.257 (1.099, 1.437) |  |
| Metformin |  |  |  | .002 |  | .002 |
| No |  |  | 1 (Ref.) |  | 1 (Ref.) |  |
| Yes |  |  | 0.857 (0.777, 0.945) |  | 0.858 (0.778, 0.947) |  |
| Meglitinide |  |  |  | .098 |  | .097 |
| No |  |  | 1 (Ref.) |  | 1 (Ref.) |  |
| Yes |  |  | 1.252 (0.959, 1.633) |  | 1.262 (0.959, 1.662) |  |
| Thiazolidinedione |  |  |  | .213 |  | .277 |
| No |  |  | 1 (Ref.) |  | 1 (Ref.) |  |
| Yes |  |  | 0.899 (0.760, 1.063) |  | 0.906 (0.757, 1.083) |  |
| DPP-4 inhibitor |  |  |  | .779 |  | .885 |
| No |  |  | 1 (Ref.) |  | 1 (Ref.) |  |
| Yes |  |  | 0.979 (0.843, 1.136) |  | 0.988 (0.836, 1.166) |  |
| Alpha -Glucosidase inhibitor |  |  |  | .615 |  | .824 |
| No |  |  | 1 (Ref.) |  | 1 (Ref.) |  |
| Yes |  |  | 0.966 (0.846, 1.104) |  | 0.980 (0.822, 1.169) |  |
| Sulfonylurea |  |  |  | .200 |  | .258 |
| No |  |  | 1 (Ref.) |  | 1 (Ref.) |  |
| Yes |  |  | 0.937 (0.849, 1.035) |  | 0.941 (0.847, 1.045) |  |

*BMI,* body mass index; *CI*, confidence interval; *CKD*, chronic kidney disease; *DM*, Diabetes mellitus; *DPP-4*, inhibitor dipeptidyl peptidase 4 inhibitors; *FBG*, fasting blood glucose; *HDL-C*, high density lipoprotein cholesterol; *HR*, hazard ratio; *LDL-C*, low density lipoprotein cholesterol; *OHA*, oral hypoglycemic agent; *PY*, pack-years; *TG,* triglycerides.

^a^Model 1 was adjusted for age, sex, income, BMI, abdominal obesity, smoking, drinking, exercise, hypertension, dyslipidemia, FBG, HDL-C, LDL-C, TG, CKD, history of stroke, DM duration, number of OHA.

^b^Model 2 was adjusted for all variables in Model 1, excluding the number of OHA, and adding Metformin, Meglitinide, TZD, DPP-4, alpha-glucosidase inhibitor, and Sulfonylurea.

^c^Model 3 was adjusted for all variables in Model 2, and adding number of OHA.

**Supplementary Table VII.** Risk of thoracic aortic aneurysm according to specified risk factors

|  | **Model 1^a^** | ***P* value** | **Model 2 ^b^** | ***P* value** | **Model 3^c^** | ***P* value** |
| --- | --- | --- | --- | --- | --- | --- |
| Age, year | 1.065 (1.059, 1.071) | <.001 | 1.065 (1.059, 1.071) | <.001 | 1.065 (1.059, 1.071) | <.001 |
| Sex |  | <.001 |  | <.001 |  | <.001 |
| Male | 1.542 (1.345, 1.768) |  | 1.540 (1.343, 1.766) |  | 1.542 (1.345, 1.768) |  |
| Female | 1 (Ref.) |  | 1 (Ref.) |  | 1 (Ref.) |  |
| Income |  | .351 |  | .347 |  | .352 |
| Q2-4 | 1 (Ref.) |  | 1 (Ref.) |  | 1 (Ref.) |  |
| Q1 | 0.944 (0.836, 1.066) |  | 0.943 (0.836, 1.065) |  | 0.944 (0.836, 1.066) |  |
| BMI, kg/m2 |  | .052 |  | .055 |  | .054 |
| <18.5 | 0.867 (0.558, 1.346) |  | 0.866 (0.558, 1.345) |  | 0.868 (0.559, 1.347) |  |
| <23 | 1 (Ref.) |  | 1 (Ref.) |  | 1 (Ref.) |  |
| <25 | 0.863 (0.748, 0.995) |  | 0.863 (0.749, 0.996) |  | 0.862 (0.748, 0.995) |  |
| <30 | 0.872 (0.752, 1.011) |  | 0.872 (0.752, 1.011) |  | 0.870 (0.750, 1.009) |  |
| ≥30 | 1.068 (0.852, 1.338) |  | 1.066 (0.850, 1.336) |  | 1.062 (0.847, 1.332) |  |
| Abdominal obesity |  | <.001 |  | <.001 |  | <.001 |
| No | 1 (Ref.) |  | 1 (Ref.) |  | 1 (Ref.) |  |
| Yes | 1.337 (1.183, 1.512) |  | 1.336 (1.181, 1.510) |  | 1.336 (1.181, 1.510) |  |
| Smoking |  | <.001 |  | <.001 |  | <.001 |
| Non | 1 (Ref.) |  | 1 (Ref.) |  | 1 (Ref.) |  |
| Ex & <20 PY | 1.026 (0.842, 1.252) |  | 1.028 (0.843, 1.253) |  | 1.027 (0.843, 1.253) |  |
| Ex & ≥20 PY | 1.530 (1.299, 1.800) |  | 1.529 (1.299, 1.800) |  | 1.528 (1.298, 1.798) |  |
| Current & <20 PY | 1.467 (1.198, 1.797) |  | 1.466 (1.197, 1.795) |  | 1.467 (1.198, 1.796) |  |
| Current & ≥20 PY | 1.646 (1.399, 1.938) |  | 1.643 (1.396, 1.934) |  | 1.643 (1.396, 1.934) |  |
| Drinking |  | .922 |  | .921 |  | .924 |
| Non | 1 (Ref.) |  | 1 (Ref.) |  | 1 (Ref.) |  |
| Mild | 1.003 (0.885, 1.137) |  | 1.004 (0.886, 1.138) |  | 1.005 (0.886, 1.139) |  |
| Heavy | 0.965 (0.793, 1.174) |  | 0.965 (0.793, 1.175) |  | 0.966 (0.794, 1.176) |  |
| Regular exercise |  | .240 |  | .247 |  | .241 |
| No | 1 (Ref.) |  | 1 (Ref.) |  | 1 (Ref.) |  |
| Yes | 0.930 (0.824, 1.050) |  | 0.931 (0.825, 1.051) |  | 0.930 (0.825, 1.050) |  |
| Hypertension |  | <.001 |  | <.001 |  | <.001 |
| No | 1 (Ref.) |  | 1 (Ref.) |  | 1 (Ref.) |  |
| Yes | 1.907 (1.670, 2.178) |  | 1.908 (1.671, 2.179) |  | 1.905 (1.668, 2.175) |  |
| Dyslipidemia |  | <.001 |  | <.001 |  | <.001 |
| No | 1 (Ref.) |  | 1 (Ref.) |  | 1 (Ref.) |  |
| Yes | 1.379 (1.243, 1.531) |  | 1.378 (1.241, 1.530) |  | 1.373 (1.237, 1.525) |  |
| HDL-C, quartile (mg/dL) |  | <.001 |  | <.001 |  | <.001 |
| Q1 (<41) | 1.379 (1.190, 1.597) |  | 1.378 (1.189, 1.596) |  | 1.376 (1.188, 1.594) |  |
| Q2 (42-49) | 1.204 (1.042, 1.390) |  | 1.204 (1.042, 1.390) |  | 1.202 (1.041, 1.388) |  |
| Q3 (50-58) | 1.198 (1.037, 1.385) |  | 1.199 (1.037, 1.385) |  | 1.197 (1.036, 1.384) |  |
| Q4 (≥59) | 1 (Ref.) |  | 1 (Ref.) |  | 1 (Ref.) |  |
| LDL-C, quartile (mg/dL) |  | .519 |  | .511 |  | .525 |
| Q1 (<84) | 1 (Ref.) |  | 1 (Ref.) |  | 1 (Ref.) |  |
| Q2 (85-109) | 0.927 (0.812, 1.058) |  | 0.927 (0.812, 1.058) |  | 0.926 (0.811, 1.058) |  |
| Q3 (110-135) | 0.965 (0.841, 1.108) |  | 0.964 (0.840, 1.107) |  | 0.965 (0.840, 1.108) |  |
| Q4 (≥136) | 0.909 (0.792, 1.044) |  | 0.908 (0.790, 1.043) |  | 0.910 (0.792, 1.046) |  |
| TG, quartile (mg/dL) |  | .006 |  | .006 |  | .006 |
| Q1 (<98) | 1 (Ref.) |  | 1 (Ref.) |  | 1 (Ref.) |  |
| Q2 (99-141) | 0.966 (0.846, 1.104) |  | 0.966 (0.846, 1.104) |  | 0.967 (0.847, 1.105) |  |
| Q3 (142-206) | 0.837 (0.728, 0.963) |  | 0.837 (0.728, 0.963) |  | 0.839 (0.729, 0.965) |  |
| Q4 (≥207) | 0.801 (0.691, 0.929) |  | 0.800 (0.690, 0.928) |  | 0.802 (0.692, 0.931) |  |
| FBG, mg/dL |  | .010 |  | .008 |  | .016 |
| ≤130 | 1 (Ref.) |  | 1 (Ref.) |  | 1 (Ref.) |  |
| >130 | 0.879 (0.797, 0.970) |  | 0.874 (0.792, 0.965) |  | 0.884 (0.800, 0.977) |  |
| CKD |  | <.001 |  | <.001 |  | <.001 |
| No | 1 (Ref.) |  | 1 (Ref.) |  | 1 (Ref.) |  |
| Yes | 1.460 (1.296, 1.643) |  | 1.460 (1.296, 1.644) |  | 1.460 (1.297, 1.645) |  |
| History of stroke |  | <.001 |  | <.001 |  | <.001 |
| No | 1 (Ref.) |  | 1 (Ref.) |  | 1 (Ref.) |  |
| Yes | 1.740 (1.407, 2.151) |  | 1.739 (1.407, 2.150) |  | 1.740 (1.407, 2.151) |  |
| Number of OHAs ≥3 |  | .131 |  |  |  | .039 |
| <3 | 1 (Ref.) |  |  |  | 1 (Ref.) |  |
| ≥3 | 0.896 (0.777, 1.033) |  |  |  | 0.790 (0.632, 0.988) |  |
| DM duration, year | 0.962 (0.946, 0.978) | <.001 | 0.959 (0.940, 0.977) | <.001 | 0.956 (0.938, 0.975) | <.001 |
| Insulin |  | .006 |  | .005 |  | .005 |
| No | 1 (Ref.) |  | 1 (Ref.) |  | 1 (Ref.) |  |
| Yes | 1.245 (1.065, 1.456) |  | 1.252 (1.070, 1.465) |  | 1.256 (1.073, 1.470) |  |
| Metformin |  |  |  | .515 |  | .751 |
| No |  |  | 1 (Ref.) |  | 1 (Ref.) |  |
| Yes |  |  | 0.963 (0.858, 1.080) |  | 0.982 (0.874, 1.102) |  |
| Meglitinide |  |  |  | .541 |  | .879 |
| No |  |  | 1 (Ref.) |  | 1 (Ref.) |  |
| Yes |  |  | 0.894 (0.623, 1.282) |  | 0.972 (0.672, 1.406) |  |
| Thiazolidinedione |  |  |  | .755 |  | .298 |
| No |  |  | 1 (Ref.) |  | 1 (Ref.) |  |
| Yes |  |  | 1.030 (0.855, 1.242) |  | 1.112 (0.911, 1.357) |  |
| DPP-4 inhibitor |  |  |  | .683 |  | .193 |
| No |  |  | 1 (Ref.) |  | 1 (Ref.) |  |
| Yes |  |  | 1.036 (0.874, 1.228) |  | 1.134 (0.938, 1.371) |  |
| Alpha -Glucosidase inhibitor |  |  |  | .629 |  | .323 |
| No |  |  | 1 (Ref.) |  | 1 (Ref.) |  |
| Yes |  |  | 0.962 (0.823, 1.125) |  | 1.108 (0.904, 1.359) |  |
| Sulfonylurea |  |  |  | .632 |  | .255 |
| No |  |  | 1 (Ref.) |  | 1 (Ref.) |  |
| Yes |  |  | 1.029 (0.916, 1.156) |  | 1.074 (0.950, 1.213) |  |

*BMI,* body mass index; *CI,* confidence interval; *CKD,* chronic kidney disease; *DM,* Diabetes mellitus; *DPP-4,* inhibitor dipeptidyl peptidase 4 inhibitors; *FBG,* fasting blood glucose; *HDL-C,* high density lipoprotein cholesterol; *HR,* hazard ratio; *LDL-C,* low density lipoprotein cholesterol; *OHA,* oral hypoglycemic agent; *PY,* pack-years; *TG,* triglycerides.

^a^Model 1 was adjusted for age, sex, income, BMI, abdominal obesity, smoking, drinking, exercise, hypertension, dyslipidemia, FBG, HDL-C, LDL-C, TG, CKD, history of stroke, DM duration, number of OHA.

^b^Model 2 was adjusted for all variables in Model 1, excluding the number of OHA, and adding Metformin, Meglitinide, TZD, DPP-4, alpha-glucosidase inhibitor, and Sulfonylurea.

^c^Model 3 was adjusted for all variables in Model 2, and adding number of OHA.

**Supplementary Table VIII.** Risk of thoracoabdominal aortic aneurysm according to specified risk factors

|  | **Model 1^a^** | ***P* value** | **Model 2 ^b^** | ***P* value** | **Model 3^c^** | ***P* value** |
| --- | --- | --- | --- | --- | --- | --- |
| Age, year | 1.067 (1.058, 1.075) | <.001 | 1.067 (1.058, 1.075) | <.001 | 1.067 (1.058, 1.075) | <.001 |
| Sex |  | <.001 |  | <.001 |  | <.001 |
| Male | 1.759 (1.431, 2.163) |  | 1.756 (1.429, 2.159) |  | 1.758 (1.430, 2.161) |  |
| Female | 1 (Ref.) |  | 1 (Ref.) |  | 1 (Ref.) |  |
| Income |  | .029 |  | .03 |  | .03 |
| Q2-4 | 1 (Ref.) |  | 1 (Ref.) |  | 1 (Ref.) |  |
| Q1 | 1.203 (1.019, 1.421) |  | 1.203 (1.019, 1.421) |  | 1.203 (1.019, 1.421) |  |
| BMI, kg/m2 |  | .603 |  | .603 |  | .605 |
| <18.5 | 0.906 (0.478, 1.717) |  | 0.904 (0.477, 1.712) |  | 0.905 (0.478, 1.714) |  |
| <23 | 1 (Ref.) |  | 1 (Ref.) |  | 1 (Ref.) |  |
| <25 | 0.975 (0.792, 1.200) |  | 0.978 (0.795, 1.204) |  | 0.978 (0.794, 1.203) |  |
| <30 | 0.942 (0.756, 1.173) |  | 0.947 (0.760, 1.179) |  | 0.946 (0.759, 1.178) |  |
| ≥30 | 1.182 (0.841, 1.659) |  | 1.189 (0.846, 1.670) |  | 1.187 (0.845, 1.667) |  |
| Abdominal obesity |  | .008 |  | .008 |  | .008 |
| No | 1 (Ref.) |  | 1 (Ref.) |  | 1 (Ref.) |  |
| Yes | 1.274 (1.064, 1.526) |  | 1.274 (1.064, 1.526) |  | 1.274 (1.064, 1.526) |  |
| Smoking |  | <.001 |  | <.001 |  | <.001 |
| Non | 1 (Ref.) |  | 1 (Ref.) |  | 1 (Ref.) |  |
| Ex & <20 PY | 1.321 (0.998, 1.749) |  | 1.323 (1.000, 1.752) |  | 1.323 (1.000, 1.752) |  |
| Ex & ≥20 PY | 1.636 (1.278, 2.095) |  | 1.637 (1.279, 2.096) |  | 1.636 (1.278, 2.095) |  |
| Current & <20 PY | 2.005 (1.518, 2.649) |  | 2.004 (1.517, 2.647) |  | 2.005 (1.518, 2.648) |  |
| Current & ≥20 PY | 2.283 (1.815, 2.872) |  | 2.283 (1.815, 2.873) |  | 2.283 (1.815, 2.872) |  |
| Drinking |  | .02 |  | .02 |  | .02 |
| Non | 1 (Ref.) |  | 1 (Ref.) |  | 1 (Ref.) |  |
| Mild | 0.898 (0.750, 1.074) |  | 0.898 (0.750, 1.074) |  | 0.898 (0.751, 1.074) |  |
| Heavy | 0.645 (0.473, 0.878) |  | 0.644 (0.473, 0.878) |  | 0.645 (0.473, 0.879) |  |
| Regular exercise |  | .265 |  | .271 |  | .268 |
| No | 1 (Ref.) |  | 1 (Ref.) |  | 1 (Ref.) |  |
| Yes | 0.903 (0.755, 1.080) |  | 0.904 (0.756, 1.082) |  | 0.904 (0.756, 1.081) |  |
| Hypertension |  | <.001 |  | <.001 |  | <.001 |
| No | 1 (Ref.) |  | 1 (Ref.) |  | 1 (Ref.) |  |
| Yes | 1.481 (1.237, 1.772) |  | 1.483 (1.239, 1.775) |  | 1.481 (1.238, 1.773) |  |
| Dyslipidemia |  | .01 |  | .01 |  | .01 |
| No | 1 (Ref.) |  | 1 (Ref.) |  | 1 (Ref.) |  |
| Yes | 1.225 (1.051, 1.428) |  | 1.227 (1.052, 1.433) |  | 1.225 (1.049, 1.430) |  |
| HDL-C, quartile (mg/dL) |  | .032 |  | .034 |  | .034 |
| Q1 (<41) | 1.214 (0.980, 1.504) |  | 1.212 (0.978, 1.502) |  | 1.211 (0.977, 1.500) |  |
| Q2 (42-49) | 1.190 (0.969, 1.462) |  | 1.190 (0.969, 1.463) |  | 1.189 (0.968, 1.461) |  |
| Q3 (50-58) | 0.927 (0.744, 1.155) |  | 0.927 (0.744, 1.155) |  | 0.927 (0.743, 1.155) |  |
| Q4 (≥59) | 1 (Ref.) |  | 1 (Ref.) |  | 1 (Ref.) |  |
| LDL-C, quartile (mg/dL) |  | .462 |  | .471 |  | .466 |
| Q1 (<84) | 1 (Ref.) |  | 1 (Ref.) |  | 1 (Ref.) |  |
| Q2 (85-109) | 1.122 (0.918, 1.372) |  | 1.122 (0.918, 1.372) |  | 1.122 (0.918, 1.372) |  |
| Q3 (110-135) | 1.162 (0.943, 1.430) |  | 1.161 (0.943, 1.430) |  | 1.162 (0.943, 1.430) |  |
| Q4 (≥136) | 1.151 (0.935, 1.417) |  | 1.149 (0.933, 1.415) |  | 1.150 (0.934, 1.417) |  |
| TG, quartile (mg/dL) |  | .126 |  | .125 |  | .127 |
| Q1 (<98) | 1 (Ref.) |  | 1 (Ref.) |  | 1 (Ref.) |  |
| Q2 (99-141) | 1.138 (0.932, 1.389) |  | 1.139 (0.933, 1.390) |  | 1.139 (0.933, 1.391) |  |
| Q3 (142-206) | 1.000 (0.812, 1.232) |  | 1.001 (0.812, 1.233) |  | 1.002 (0.813, 1.234) |  |
| Q4 (≥207) | 0.887 (0.708, 1.111) |  | 0.888 (0.708, 1.112) |  | 0.889 (0.709, 1.114) |  |
| FBG, mg/dL |  | .006 |  | .004 |  | .006 |
| ≤130 | 1 (Ref.) |  | 1 (Ref.) |  | 1 (Ref.) |  |
| >130 | 0.814 (0.704, 0.941) |  | 0.808 (0.698, 0.935) |  | 0.814 (0.703, 0.943) |  |
| CKD |  | .001 |  | .001 |  | .001 |
| No | 1 (Ref.) |  | 1 (Ref.) |  | 1 (Ref.) |  |
| Yes | 1.376 (1.149, 1.649) |  | 1.370 (1.143, 1.642) |  | 1.370 (1.143, 1.643) |  |
| History of stroke |  | <.001 |  | <.001 |  | <.001 |
| No | 1 (Ref.) |  | 1 (Ref.) |  | 1 (Ref.) |  |
| Yes | 1.858 (1.353, 2.551) |  | 1.854 (1.350, 2.545) |  | 1.854 (1.350, 2.546) |  |
| Number of OHAs ≥3 |  | .537 |  |  |  | .384 |
| <3 | 1 (Ref.) |  |  |  | 1 (Ref.) |  |
| ≥3 | 1.067 (0.869, 1.310) |  |  |  | 0.865 (0.625, 1.199) |  |
| DM duration, year | 0.955 (0.931, 0.978) | <.001 | 0.953 (0.925, 0.981) | .001 | 0.951 (0.924, 0.979) | <.001 |
| Insulin |  | .113 |  | .122 |  | .119 |
| No | 1 (Ref.) |  | 1 (Ref.) |  | 1 (Ref.) |  |
| Yes | 1.212 (0.955, 1.536) |  | 1.208 (0.951, 1.534) |  | 1.209 (0.952, 1.536) |  |
| Metformin |  |  |  | .427 |  | .522 |
| No |  |  | 1 (Ref.) |  | 1 (Ref.) |  |
| Yes |  |  | 0.933 (0.786, 1.107) |  | 0.945 (0.795, 1.124) |  |
| Meglitinide |  |  |  | .569 |  | .701 |
| No |  |  | 1 (Ref.) |  | 1 (Ref.) |  |
| Yes |  |  | 0.851 (0.488, 1.483) |  | 0.895 (0.507, 1.578) |  |
| Thiazolidinedione |  |  |  | .897 |  | .673 |
| No |  |  | 1 (Ref.) |  | 1 (Ref.) |  |
| Yes |  |  | 1.019 (0.769, 1.350) |  | 1.066 (0.791, 1.438) |  |
| DPP-4 inhibitor |  |  |  | .407 |  | .259 |
| No |  |  | 1 (Ref.) |  | 1 (Ref.) |  |
| Yes |  |  | 1.113 (0.865, 1.431) |  | 1.174 (0.889, 1.552) |  |
| Alpha -Glucosidase inhibitor |  |  |  | .051 |  | .040 |
| No |  |  | 1 (Ref.) |  | 1 (Ref.) |  |
| Yes |  |  | 1.244 (0.999, 1.548) |  | 1.356 (1.014, 1.815) |  |
| Sulfonylurea |  |  |  | .875 |  | .663 |
| No |  |  | 1 (Ref.) |  | 1 (Ref.) |  |
| Yes |  |  | 1.014 (0.852, 1.207) |  | 1.042 (0.867, 1.252) |  |

*BMI,* body mass index; *CI,* confidence interval; *CKD,* chronic kidney disease; *DM,* Diabetes mellitus; *DPP-4,* inhibitor dipeptidyl peptidase 4 inhibitors; *FBG,* fasting blood glucose; *HDL-C,* high density lipoprotein cholesterol; *HR,* hazard ratio; *LDL-C,* low density lipoprotein cholesterol; *OHA,* oral hypoglycemic agent; *PY*, pack-years; *TG,* triglycerides.

^a^Model 1 was adjusted for age, sex, income, BMI, abdominal obesity, smoking, drinking, exercise, hypertension, dyslipidemia, FBG, HDL-C, LDL-C, TG, CKD, history of stroke, DM duration, number of OHA.

^b^Model 2 was adjusted for all variables in Model 1, excluding the number of OHA, and adding Metformin, Meglitinide, TZD, DPP-4, alpha-glucosidase inhibitor, and Sulfonylurea.

^c^Model 3 was adjusted for all variables in Model 2, and adding number of OHA.

**Supplementary Table VIIII.** Interaction analysis according to age and sex

|  |  | **N** | **Event** | **Duration** | **IR, 1000 PY** | **N** | **Univariate model** | ***P* for interaction** | **Model 1^a^** | ***P* for interaction** | **Model 2^b^** | ***P* for interaction** |
| --- | --- | --- | --- | --- | --- | --- | --- | --- | --- | --- | --- | --- |
| TAAD | Age <65,  Male | 995,120 | 1,404 | 7,849,691.27 | 0.18 | 1,404 | 1.480  (1.342, 1.633) | .867 | 1.377  (1.191, 1.592) | .5241 | 1.378  (1.192, 1.593) | .524 |
|  | Age <65, Female | 572,184 | 556 | 4,608,134.70 | 0.12 | 556 | 1 (Ref.) |  | 1 (Ref.) |  | 1 (Ref.) |  |
|  | Age ≥65,  Male | 352,159 | 1,387 | 2,503,194.16 | 0.55 | 1,387 | 1.464  (1.355, 1.583) |  | 1.448  (1.293, 1.623) |  | 1.449  (1.293, 1.624) |  |
|  | Age ≥65, Female | 407,368 | 1,165 | 3,085,756.48 | 0.38 | 1,165 | 1 (Ref.) |  | 1 (Ref.) |  | 1 (Ref.) |  |
| TAD | Age <65,  Male | 995,120 | 753 | 7,852,308.27 | 0.10 | 753 | 1.367  (1.200, 1.558) | .294 | 1.127  (0.925, 1.373) | .6094 | 1.127  (0.925, 1.373) | .609 |
|  | Age <65, Female | 572,184 | 323 | 4,609,142.11 | 0.07 | 323 | 1 (Ref.) |  | 1 (Ref.) |  | 1 (Ref.) |  |
|  | Age ≥65,  Male | 352,159 | 660 | 2,505,703.04 | 0.26 | 660 | 1.249  (1.121, 1.392) |  | 1.191  (1.013, 1.399) |  | 1.191  (1.013, 1.399) |  |
|  | Age ≥65, Female | 407,368 | 651 | 3,087,602.38 | 0.21 | 651 | 1 (Ref.) |  | 1 (Ref.) |  | 1 (Ref.) |  |
| TAA | Age <65,  Male | 995,120 | 499 | 7,853,048.92 | 0.06 | 499 | 1.641  (1.383, 1.947) | .782 | 1.676  (1.309, 2.145) | .749 | 1.677  (1.310, 2.146) | .750 |
|  | Age <65, Female | 572,184 | 178 | 4,609,560.68 | 0.04 | 178 | 1 (Ref.) |  | 1 (Ref.) |  | 1 (Ref.) |  |
|  | Age ≥65,  Male | 352,159 | 568 | 2,505,784.31 | 0.23 | 568 | 1.592  (1.405, 1.804) |  | 1.605  (1.338, 1.927) |  | 1.607  (1.339, 1.928) |  |
|  | Age ≥65, Female | 407,368 | 437 | 3,088,134.11 | 0.14 | 437 | 1 (Ref.) |  | 1 (Ref.) |  | 1 (Ref.) |  |
| TAAA | Age <65,  Male | 995,120 | 231 | 7,854,341.71 | 0.03 | 231 | 1.575  (1.230, 2.018) | .027 | 1.915  (1.324, 2.770) | .5377 | 1.913  (1.323, 2.767) | .538 |
|  | Age <65, Female | 572,184 | 86 | 4,609,998.18 | 0.02 | 86 | 1 (Ref.) |  | 1 (Ref.) |  | 1 (Ref.) |  |
|  | Age ≥65,  Male | 352,159 | 294 | 2,506,857.24 | 0.12 | 294 | 2.244  (1.853, 2.719) |  | 2.170  (1.656, 2.844) |  | 2.167  (1.654, 2.840) |  |
|  | Age ≥65, Female | 407,368 | 162 | 3,089,266.11 | 0.05 | 162 | 1 (Ref.) |  | 1 (Ref.) |  | 1 (Ref.) |  |

*FBG,* fasting blood glucose; *IR,* incidence rate; *N,* number; *PY,* Person-Years; *TAA,* thoracic aortic aneurysm; *TAAA,* thoracoabdominal aortic aneurysm; *TAAD,* thoracic aortic aneurysm and dissection; *TAD,* thoracic aortic dissection.

^a^Model 1 was adjusted for age, sex, income, BMI, abdominal obesity, smoking, drinking, exercise, hypertension, dyslipidemia, FBG, HDL-C, LDL-C, TG, CKD, history of stroke, DM duration, Metformin, Meglitinide, TZD, DPP-4, alpha-glucosidase inhibitor, Sulfonylurea

^b^Model 2 was adjusted for age, sex, income, BMI, abdominal obesity, smoking, drinking, exercise, hypertension, dyslipidemia, FBG, HDL-C, LDL-C, TG, CKD, history of stroke, DM duration, number of OHA, Metformin, Meglitinide, TZD, DPP-4, alpha-glucosidase inhibitor, Sulfonylurea

**SUPPLEMENTARY FIGURE CAPTION**

Supplementary Figure 1. Cumulative incidence of TAAD stratified by sex.

Supplementary Figure 2. Cumulative incidence of TAAD stratified by abdominal obesity.

Supplementary Figure 3. Cumulative incidence of TAAD stratified by smoking.

Supplementary Figure 4. Cumulative incidence of TAAD stratified by alcohol intake.

Supplementary Figure 5. Cumulative incidence of TAAD stratified by hypertension.

Supplementary Figure 6. Cumulative incidence of TAAD stratified by dyslipidemia.

Supplementary Figure 7. Cumulative incidence of TAAD stratified by HDL-C.

Supplementary Figure 8. Cumulative incidence of TAAD stratified by heart failure.

Supplementary Figure 9. Cumulative incidence of TAAD stratified by coronary heart disease.

Supplementary Figure 10. Cumulative incidence of TAAD stratified by chronic obstructive pulmonary disease.
