# Supplementary figures and images for: Risk factors for thoracic aortic aneurysm and dissection among diabetic patients: a nationwide population-based study
Source: Front Cardiovasc Med. 2025 Oct 7;12:1569886. doi: 10.3389/fcvm.2025.1569886 (PMC12537662; doi:10.3389/fcvm.2025.1569886)

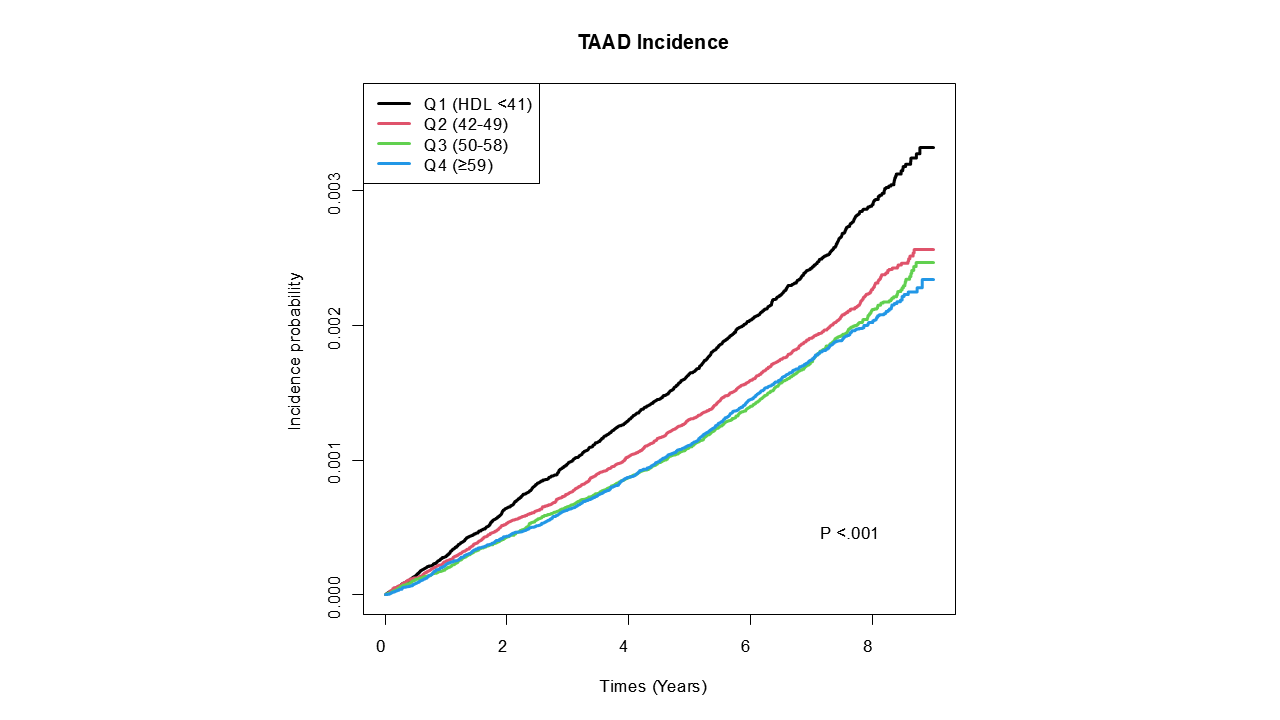

Supplement: Supplementary file 2 [file Image1.tif]

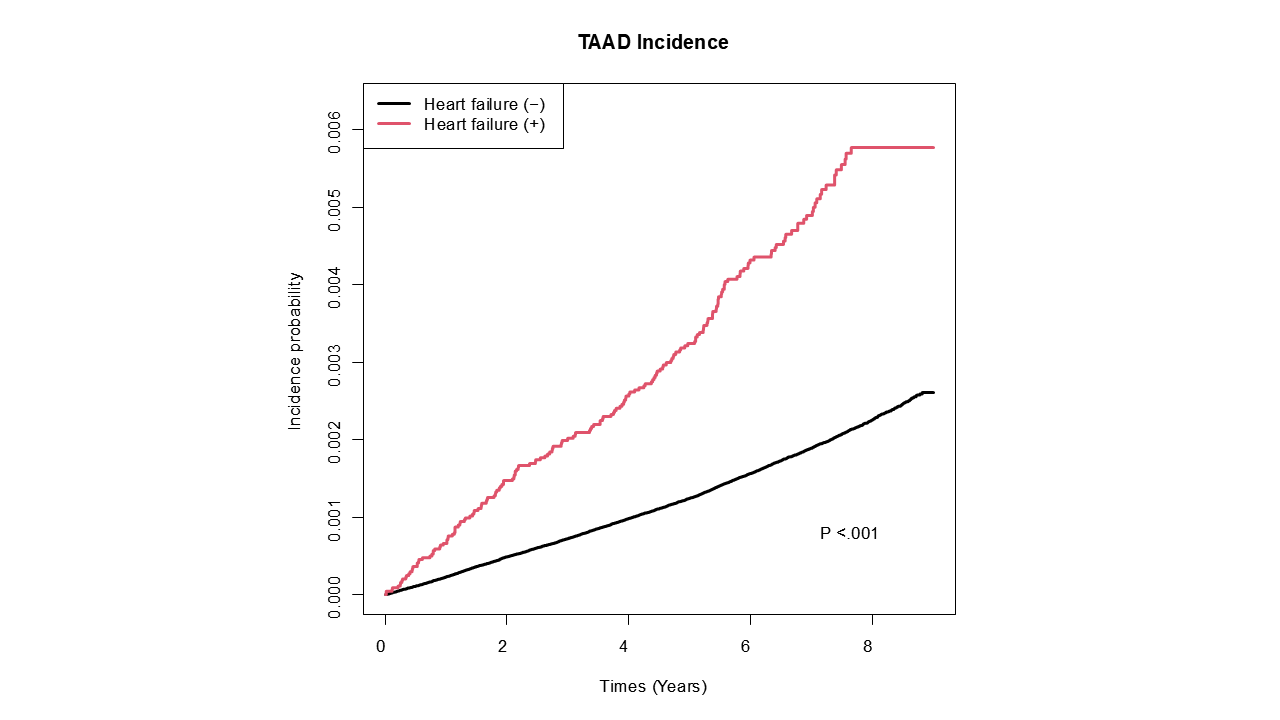

Supplement: Supplementary file 3 [file Image2.tif]

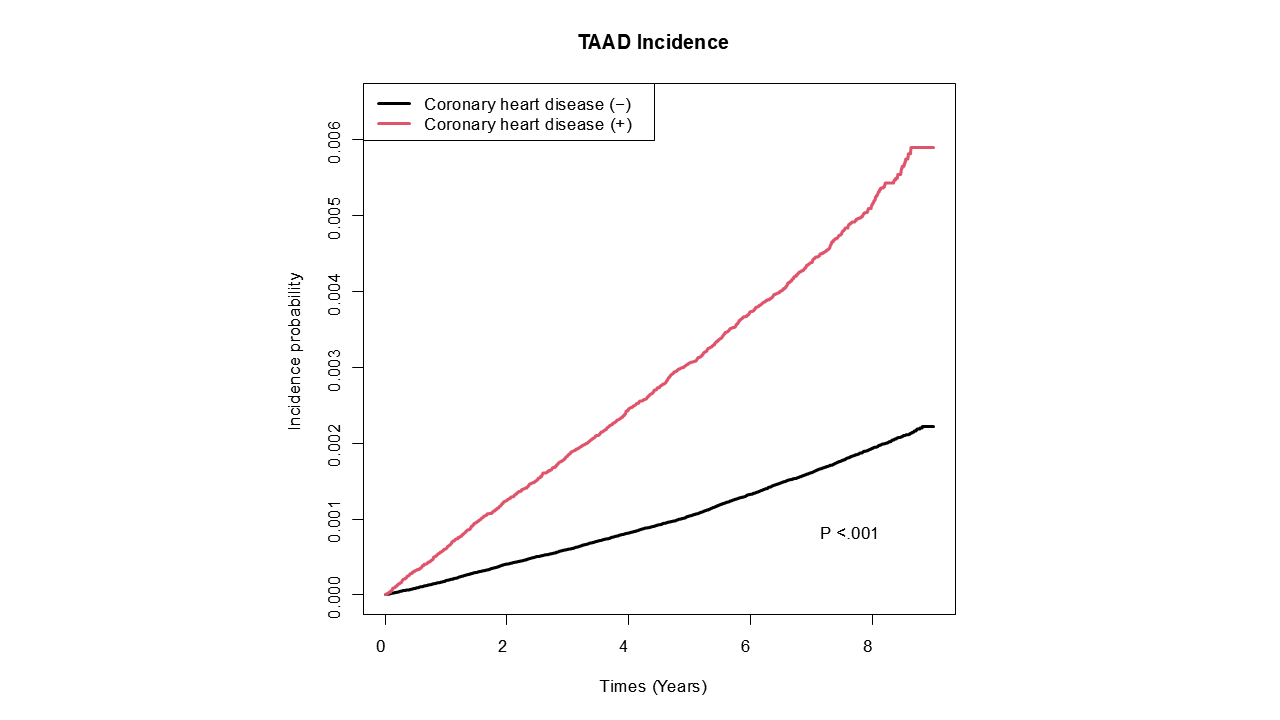

Supplement: Supplementary file 4 [file Image3.tif]

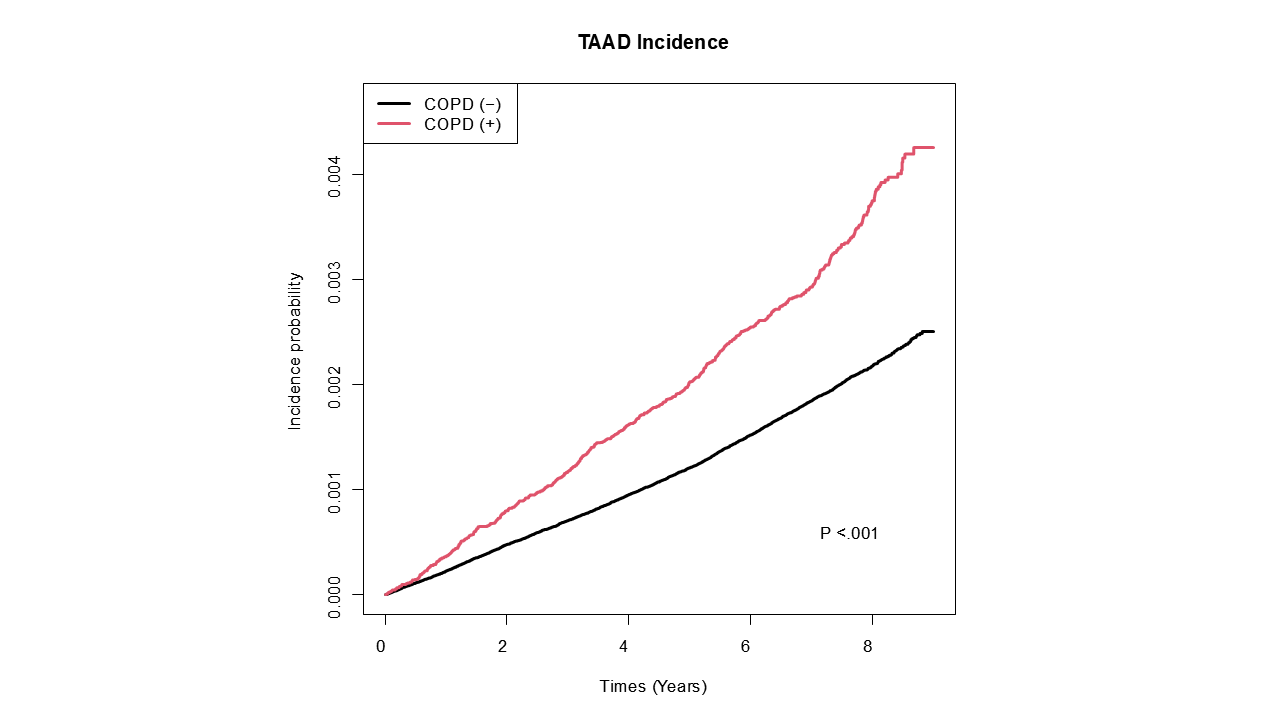

Supplement: Supplementary file 5 [file Image4.tif]

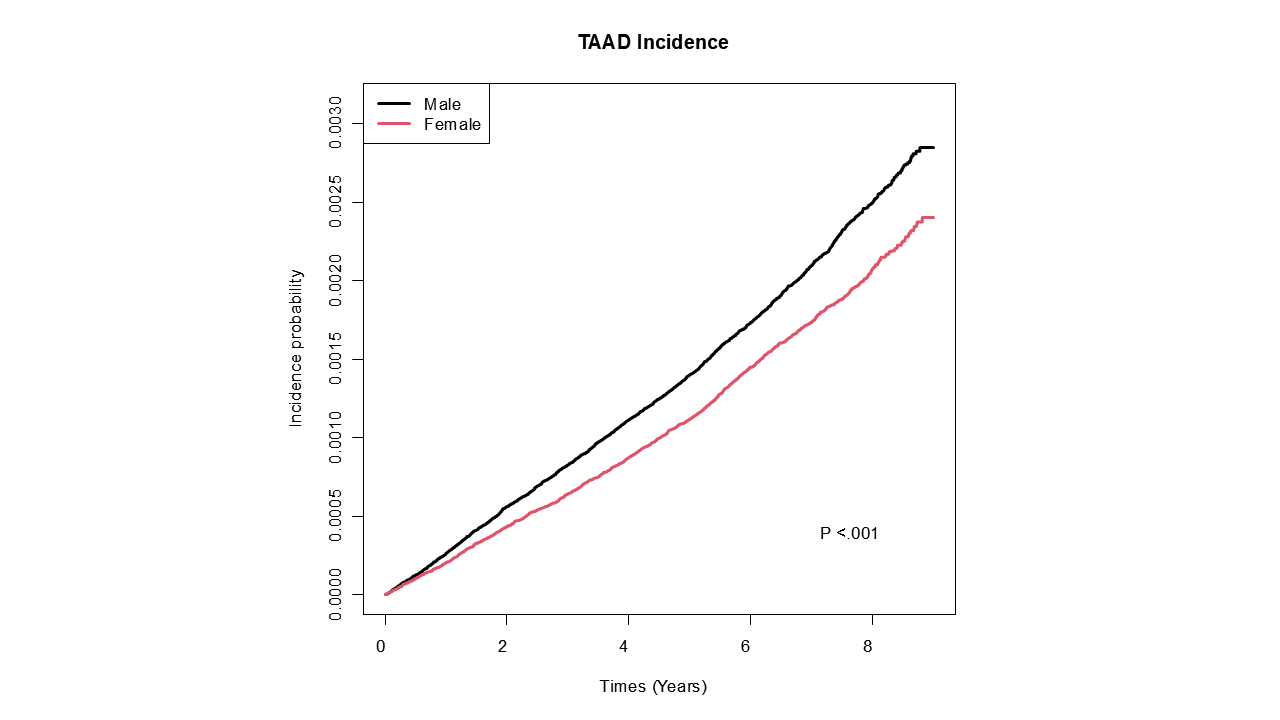

Supplement: Supplementary file 6 [file Image5.tif]

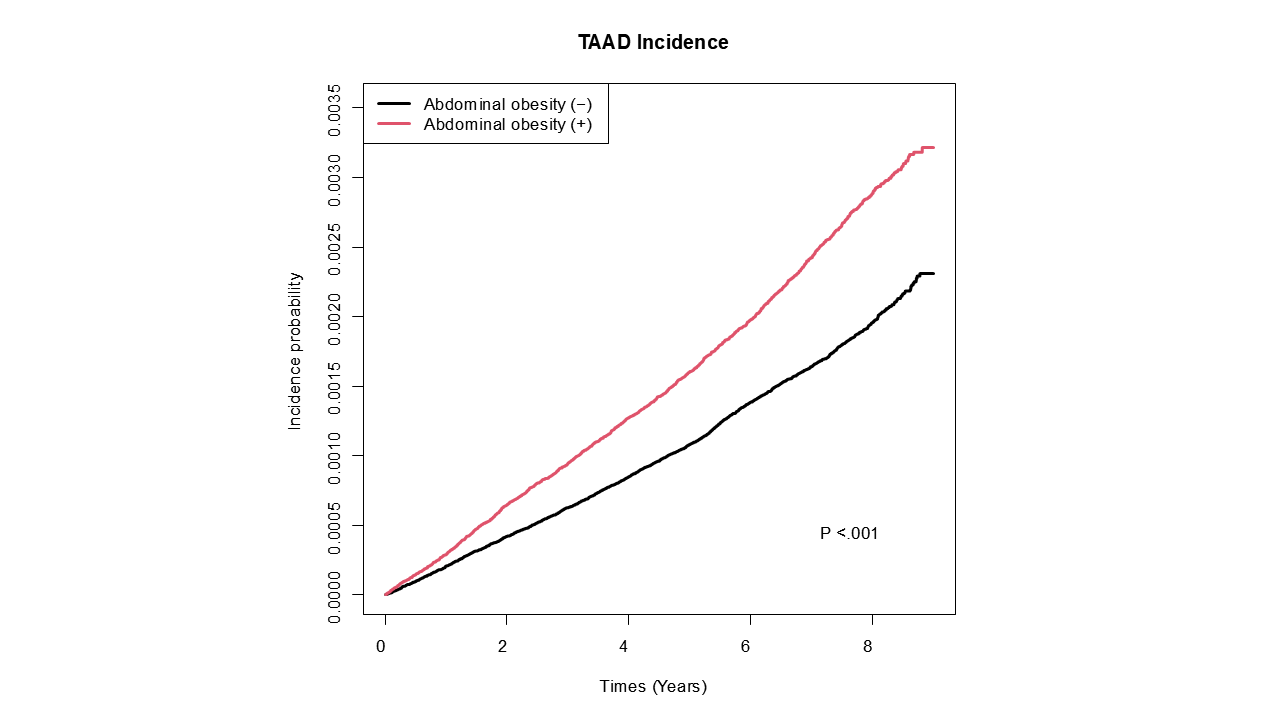

Supplement: Supplementary file 7 [file Image6.tif]

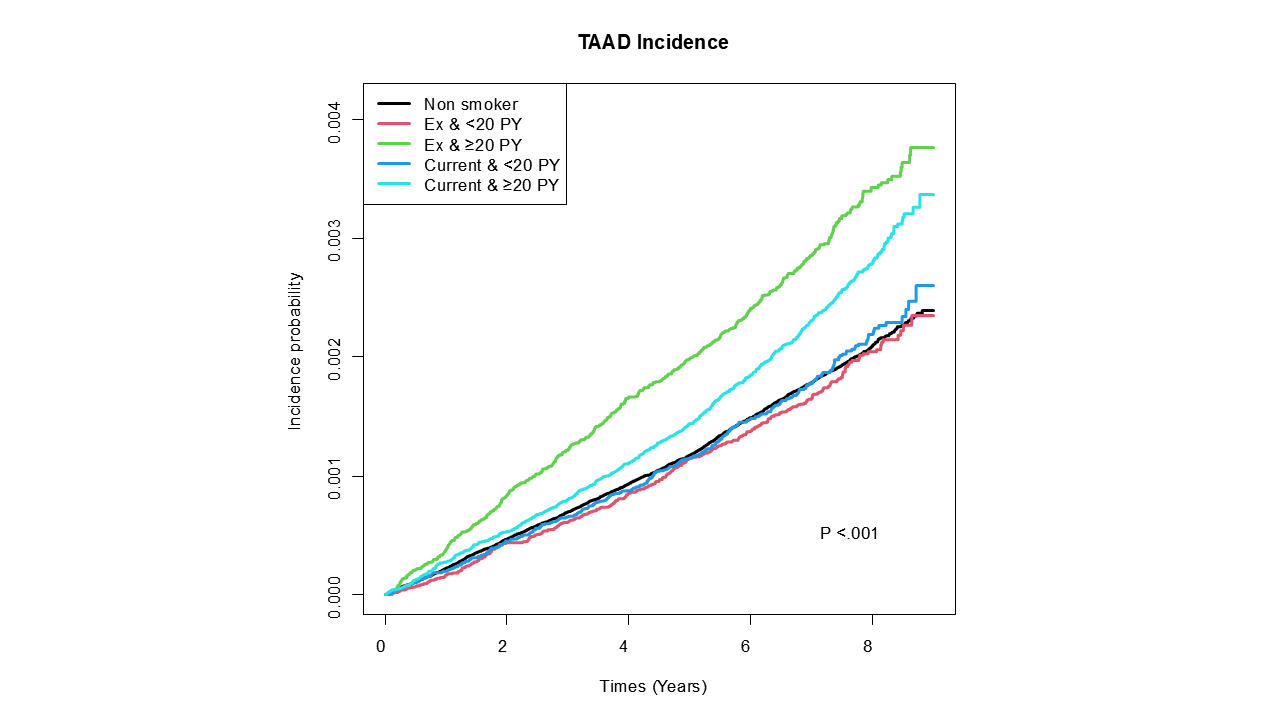

Supplement: Supplementary file 8 [file Image7.tif]

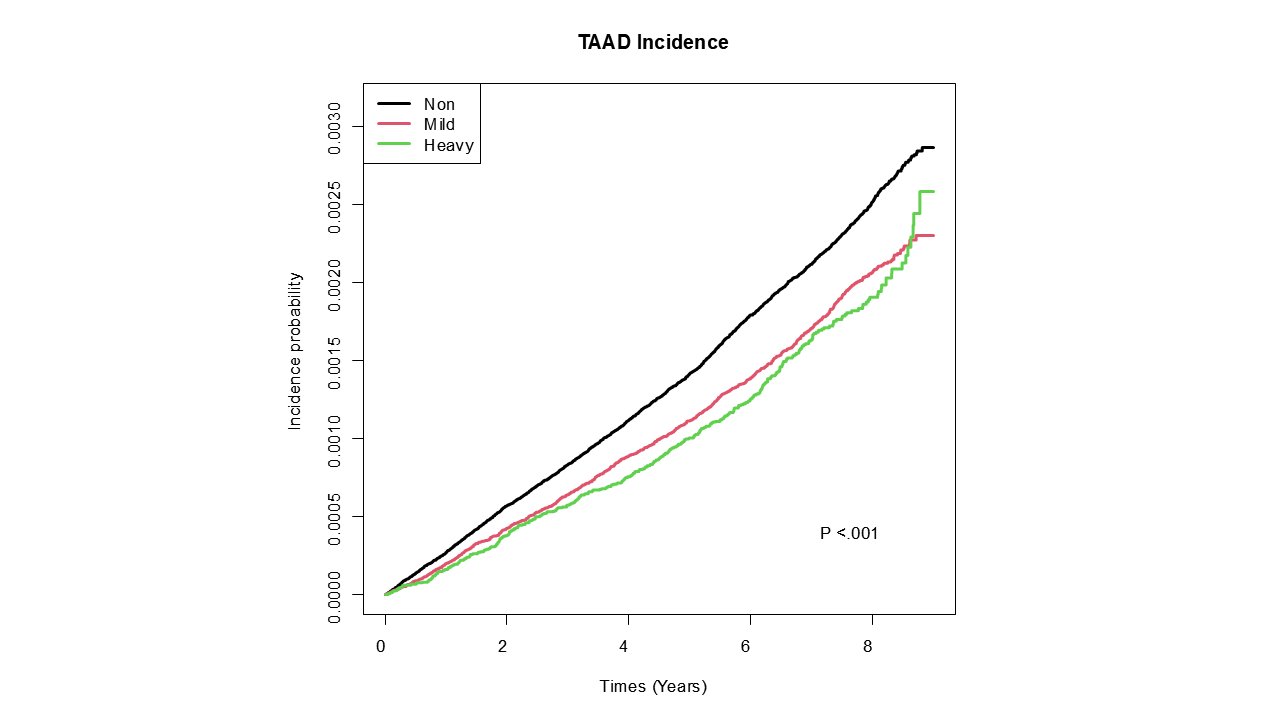

Supplement: Supplementary file 9 [file Image8.tif]

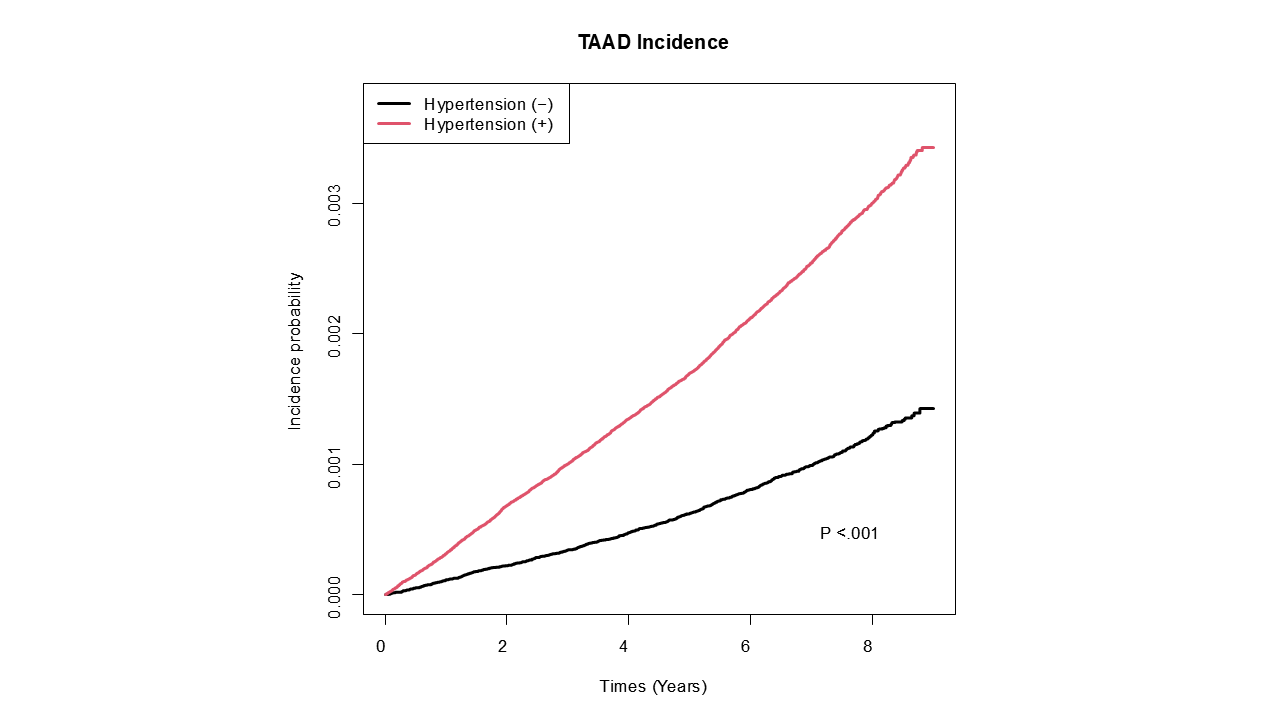

Supplement: Supplementary file 10 [file Image9.tif]

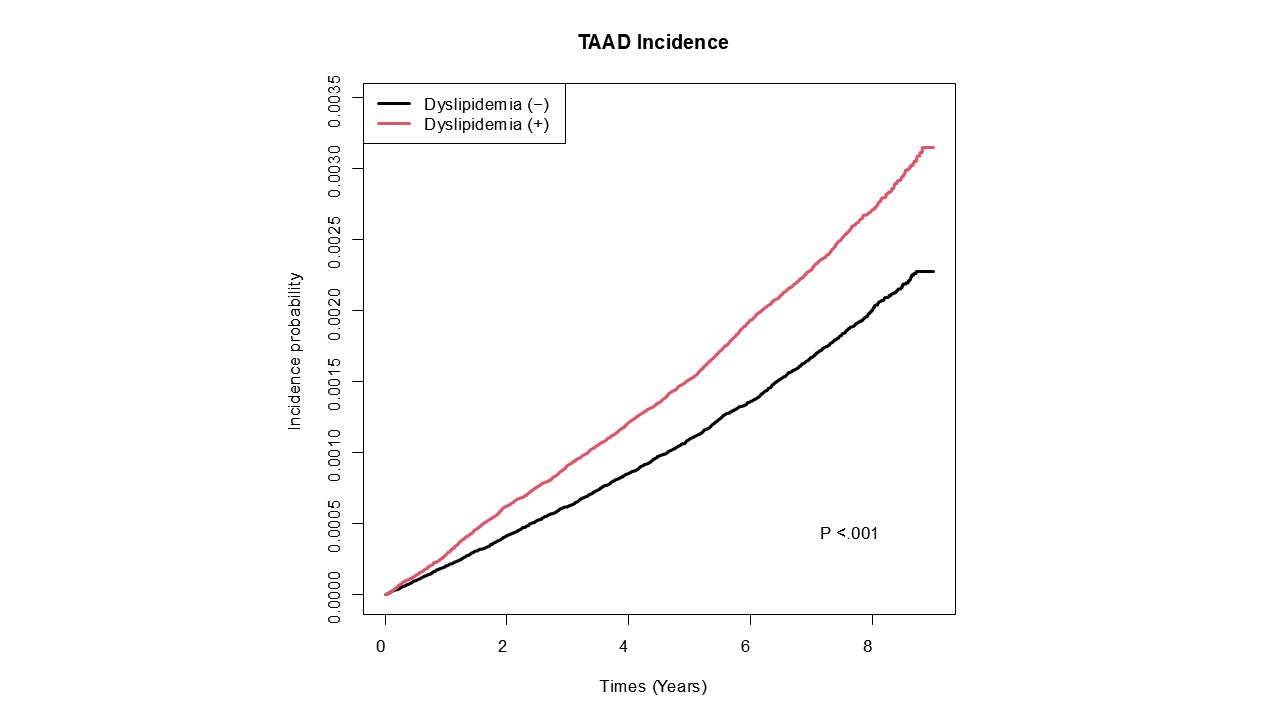

Supplement: Supplementary file 11 [file Image10.tif]
